# In-Network Processing of an Iceberg Join Query in Wireless Sensor Networks Based on 2-Way Fragment Semijoins

**DOI:** 10.3390/s150306105

**Published:** 2015-03-12

**Authors:** Hyunchul Kang

**Affiliations:** School of Computer Science and Engineering, Chung-Ang University, Seoul 156-756, Korea; E-Mail: hckang@cau.ac.kr; Tel.: 82-2-820-5306; Fax: 82-2-826-0853

**Keywords:** iceberg join query, wireless sensor networks, sensor network database, 2-way fragment semijoin, optimal sequence of 2-way fragment semijoins

## Abstract

We investigate the in-network processing of an iceberg join query in wireless sensor networks (WSNs). An iceberg join is a special type of join where only those joined tuples whose cardinality exceeds a certain threshold (called iceberg threshold) are qualified for the result. Processing such a join involves the value matching for the join predicate as well as the checking of the cardinality constraint for the iceberg threshold. In the previous scheme, the value matching is carried out as the main task for filtering non-joinable tuples while the iceberg threshold is treated as an additional constraint. We take an alternative approach, meeting the cardinality constraint first and matching values next. In this approach, with a logical fragmentation of the join operand relations on the aggregate counts of the joining attribute values, the optimal sequence of 2-way fragment semijoins is generated, where each fragment semijoin employs a Bloom filter as a synopsis of the joining attribute values. This sequence filters non-joinable tuples in an energy-efficient way in WSNs. Through implementation and a set of detailed experiments, we show that our alternative approach considerably outperforms the previous one.

## 1. Introduction

In wireless sensor networks (WSNs), the values sampled by a senor node can be modeled as a relational tuple that consists of the sensor readings as its main attributes and often of the node ID, the timestamp of the sampling, the location of the node, etc. as its auxiliary attributes [1]. Thus, for a region of WSNs, the sensor readings of the nodes deployed in the region can be modeled as a *virtual* relation physically distributed across the senor nodes in the region. In WSN applications, which include vehicle surveillance, environment monitoring, animal habitat monitoring, and climate research to name just a few, a relational *join* query can be issued against two virtual relations. For example, based on the scenario in vehicle surveillance presented in [2], let us consider the identification of the moving objects that have passed two particular regions in WSNs. In each region, the ID of a passing object is sampled and stored with the time of passage. Then, the sets of sensor readings stored in the two regions are modeled as virtual relations denoted as ${𝓡}_{0}$ and ${𝓡}_{1}$. The following join query is to retrieve the ID and time of the objects that have passed both of the two regions:

$$\begin{array}{c} \mathrm{SELECT}\;{𝓡}_{0}.ID,\;{𝓡}_{0}.timestamp,\;{𝓡}_{1}.timestamp\\ \mathrm{FROM}\;{𝓡}_{0},\;{𝓡}_{1}\\ \mathrm{WHERE}\;{𝓡}_{0}\;.ID=\;{𝓡}_{1}.ID \end{array}$$

Since a join is an important type of query in WSNs to monitor the *correlations* among the senor readings, processing of joins in WSNs has received much attention. A survey of the state-of-the-art techniques is presented in [3]. A naïve method to answer a join query, ${𝓡}_{0}$ ⋈ ${𝓡}_{1}$, in WSNs is the *external* join, whereby all the tuples of ${𝓡}_{0}$ and ${𝓡}_{1}$ are sent to the base station where the result of the join is produced. In WSNs, the power in a node is consumed the most when the node transmits data [4]. Thus, the external join is not energy-efficient, and the state-of-the-art techniques conduct *in-network* processing of joins.

**Figure 1:**
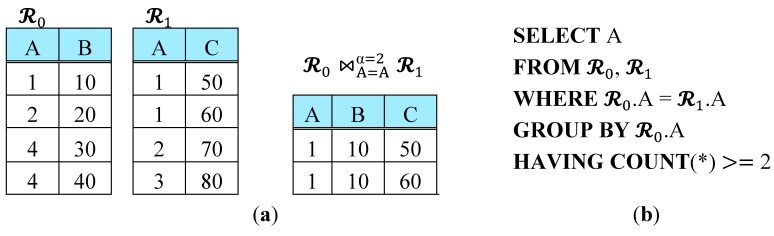
An Example of an Iceberg Join Query. (**a**) Join operand relations ${𝓡}_{0}$ and ${𝓡}_{1}$, and the result of their iceberg join with *α* = 2; (**b**) SQL expression retrieving attribute A from the iceberg join in (**a**).

In this paper, we investigate the in-network processing of a special type of equijoin query called *iceberg join* in WSNs. It is to retrieve the *frequent* patterns of correlation among the sensor readings. For a joining attribute value *v*, it contributes to the join result only if the number of joined tuples for *v* exceeds some given threshold. This join frequency threshold is called *iceberg threshold* and denoted as *α* throughout this paper. Figure 1a shows an example of iceberg join of two relations ${𝓡}_{0}$ and ${𝓡}_{1}$ with *α* = 2, denoted as ${𝓡}_{0}\;{⋈}_{\text{A}=\text{A}}^{\text{α}=2}\;{𝓡}_{1}$. A query retrieving attribute A from this iceberg join can be expressed in SQL as in Figure 1b.

In bird habitat monitoring, sensor nodes can be deployed to sample bird songs when birds are singing. From these audio samples, their fingerprints are generated, stored, and later used to recognize the bird species and to estimate their population size in certain regions [5]. For two regions of interest in WSNs, an iceberg join query can be issued to retrieve the fingerprints that are frequently sampled in both regions in studying regional correlations in bird population. Let ${𝓡}_{0}$ and ${𝓡}_{1}$ denote the virtual relations storing the fingerprints in the two regions. Then, this query can be expressed as:

$$\begin{array}{c} \mathrm{SELECT}\;{𝓡}_{0}.fingerprint\\ \mathrm{FROM}\;{𝓡}_{0},\;{𝓡}_{1}\\ \mathrm{WHERE}\;{𝓡}_{0}.fingerprint=\;{𝓡}_{1}.fingerprint\\ \mathrm{GROUP}\;\mathrm{BY}\;{𝓡}_{0}.fingerprint\\ \mathrm{HAVING}\;\mathrm{COUNT}(*)\;>=\alpha \end{array}$$

The iceberg join is an important type of query in WSNs because the frequent or prominent phenomena of interest in terms of correlations among sensor readings could be detected in a more energy-efficient way than with the conventional joins. Considering the resource constraints in WSNs and the cross-references required in join processing, the processing of the conventional types of join queries in WSNs could be too expensive [3]. The efficient processing of iceberg joins in WSNs deserves attention but so far little work has been reported. The most relevant ones include the schemes proposed in [6,7,8].

An iceberg join operation involves the checking of the *join predicate* and of the *cardinality constraint* between the tuples of the join operand relations. There could be two approaches depending on which condition of the two is the primary one to check. The primary condition is checked first, and for the tuples that satisfy it, the remaining condition is checked next. It would be reasonable to regard the join predicate as an intrinsic requirement of a join operation while treating the cardinality constraint as an additional one. In [6], such a view was taken, and a scheme called *SRJA* (Synopsis Refinement iceberg-Join Algorithm) was proposed, where a histogram-based synopsis of the joining attribute value ranges is transmitted for filtering non-joinable tuples. In [6], it was shown that SRJA significantly outperformed the baseline schemes. In this paper, we investigate an *alternative* approach where the cardinality constraint is checked first as the primary condition and then for those tuples that satisfy it, the join predicate is checked. We show that this approach is substantially superior to the other one. The contributions of this paper are as follows:

- We consider a logical *fragmentation* of join operand relations based on the aggregate counts of the joining attribute values, proposing a *2-way fragment semijoin* operation using a Bloom filter as a synopsis of the joining attribute values. In the *backward reduction* of the 2-way fragment semijoin, the false positives inherent with the Bloom filter are efficiently handled.
- We take advantage of the *Highest Count First* strategy with which efficient reduction of the join operand relation (called *Low Count Cut*) occurs, developing a dynamic programming algorithm that generates the *optimal* sequence of 2-way fragment semijoins. The Highest Count First strategy is shown to be more effective in filtering non-joinable tuples than the transmissions of the value ranges widely used in WSNs.
- Through implementation and a set of detailed experiments, we show that our approach considerably outperforms the previous one.

The rest of this paper is organized as follows: in Section 2, the problem statement is given. In Section 3, the background for our scheme is presented. In Section 4, an overview of our approach is given. In Section 5, the optimization with a dynamic programming algorithm is described. In Section 6, the performance of our scheme is compared with that of SRJA. In Section 7, related work is presented. Finally, in Section 8, the conclusions are drawn and the future work is given. Notations used in this paper are summarized in Table 1 of Section 4.

## 2. Problem Statement

An iceberg join query *Q* for two virtual relations ${𝓡}_{0}$ and ${𝓡}_{1}$ on attribute *A* in WSNs is assumed to be submitted to the base station as a continuous query modeled as a sliding window join [9]. Each evaluation of the query is conducted against a window of ${𝓡}_{0}$ and that of ${𝓡}_{1}$, and the query result is returned to the base station. Initially, the base station forwards *Q* to three sensor nodes ${\tilde{n}}_{0}$, ${\tilde{n}}_{1}$, and $\tilde{m}$. ${\tilde{n}}_{i}$ is the coordinator node of the region for ${𝓡}_{i}$, *i* = 0, 1. $\tilde{m}$ is the node located at the midpoint between ${\tilde{n}}_{0}$ and ${\tilde{n}}_{1}$, which is to take part in in-network query optimization and processing. In each region, a *routing tree* whose root is ${\tilde{n}}_{i}$ is constructed with the standard routing tree construction algorithm of [1]. ${\tilde{n}}_{i}\;$disseminates *Q* to all the sensor nodes in the region. In each region, *preprocessing* is carried out to collect the aggregate count of each joining attribute value that is sampled in the window. Each sensor node in a region generates a *node histogram*, which is a set of (value, count) pairs in the node. Then, it sends the node histogram to its parent node in the routing tree. Eventually, ${\tilde{n}}_{i}$ obtains the *region histogram*. It is a binary relation with the joining attribute *A* and the *count* attribute. In [6], this relation is called the *base histogram*. Let us denote the base histogram of region ${𝓡}_{0}$ and ${𝓡}_{1}$ as $R_{0}$ and $R_{1}$, respectively. Now the problem is to fully reduce $R_{0}$ and $R_{1}$ such that only those tuples of them that are qualified for the iceberg join remain. Let $R\prime_{0}$ and $R\prime_{1}$ respectively denote the full reduction of $R_{0}$ and $R_{1}$. Then, the final result of *Q* can be obtained by two semijoins followed by a final join: (${𝓡}_{0}$ ${⋉}_{A=A}$ $R\prime_{0}$) ${⋈}_{A=A}$ (${𝓡}_{1}{⋉}_{A=A}$ $R\prime_{1}$). Once $R\prime_{0}$ and $R\prime_{1}$ are obtained, the operations to produce the final result of *Q* are straightforward. Thus, in this paper, we deal only with the problem of optimally obtaining $R\prime_{0}$ and $R\prime_{1}$.

## 3. Background

Our scheme employs the Bloom filter [10] and a variation of the 2-way semijoin [11,12]. In Section 3.1, semijoin and 2-way semijoin operations are briefly described. In Section 3.2, an overview of Bloom filter and its theory are given. In Section 3.3, the technique of SRJA [6] is described.

### 3.1. Semijoin and 2-Way Semijoin

The semijoin was proposed in [13] as a means to reduce join operand relations in distributed databases. Given a join $R_{0}$ ${⋈}_{A=A}$ $R_{1}$, semijoin $R_{0}{⋊}_{A=A}R_{1}$ reduces $R_{1}$ such that only those tuples of $R_{1}$ joinable with $R_{0}$ remain. Assuming that $R_{0}$ and $R_{1}$ reside at different sites in distributed databases, the semijoin $R_{0}{⋊}_{A=A}R_{1}$ is executed by sending $R_{0}$ [A] to $R_{1}$ and joining the two. That is, $R_{0}{⋊}_{A=A}R_{1}$ = $R_{0}\left[\text{A}\right]\;{⋈}_{A=A}R_{1}$. $R_{1}$ is said to be fully reduced by the semijoin. Let us call $R_{0}$ the *reducer relation*, and $R_{1}$ the *reduced relation*. The size of the result of semijoin $R_{0}{⋊}_{A=A}R_{1}$ is $\left|R_{1}\right|\cdot s$, where *s* is called the semijoin selectivity and it is estimated as *|R*[A]*|/|D_A_|*, where $D_{A}$ is the domain of *A* [14].

The 2-way semijoin was investigated in [11,12,15] as an extension of the semijoin. It includes the *backward reduction* phase in addition to the forward reduction phase of the original semijoin such that both of the two join operand relations are fully reduced. Suppose semijoin $R_{0}{⋊}_{A=A}R_{1}$ reduces $R_{1}$ to $R\prime_{1}$. In the backward reduction phase of 2-way semijoin $R_{0}⋊{⋉}_{A=A}R_{1}$, $R\prime_{1}$[A] or its complement (*i.e.*, $R_{1}$[A]$\;-\;R\prime_{1}$[A]) or a bit vector indicating which value of $R_{1}$[A] is joinable and which is not is sent back. In distributed query processing, this backward reduction is often effective and can be applied to a pipelined *n*-way join [12]. In our scheme proposed in this paper, the backward reduction is efficiently merged with the handling of the false positives inherent with the Bloom filter employed in implementing a semijoin.

### 3.2. Bloom Filter

A *k*-transform Bloom filter is a bit vector of length *m* that probabilistically represents a set *S* with *k* hash functions *h*_1_(), ⋯, *h_k_*() for *k* ≥ 1 [10]. Initially, all the *m* bits are set to 0. For each value *x* in *S*, *h*_1_(*x*)-th, ⋯ , *h_k_*(*x*)-th bits of the Bloom filter are set. For a given value *y*, *y* is not in *S* if any of the *h*_1_(*y*)-th, ⋯, *h_k_*(*y*)-th bits of the Bloom filter is not 1, whereas *y* is *probably* in *S* if all those *k* bits are 1. In the latter case, a *false positive* is possible due to the possibility of the collisions in hashing. However, it is guaranteed that false negatives are not possible.

For two relations $R_{0}$ and $R_{1}$ that reside at different locations, a Bloom filter can be employed in implementing semijoin $R_{0}{⋊}_{A=A}\;R_{1}$ with possible errors. First, $R_{0}$ is scanned to construct a Bloom filter bf {$R_{0},\;$*A*} that represents $R_{0}$ [A] bf{$R_{0},\;$*A*} is sent to $R_{1}$. Then, for each value *v* of $R_{1}$.A, the membership test of *v* ∈bf{$R_{0},\;$*A*} is done to filter non-joinable tuples of $R_{1}$. None of the joinable tuples of $R_{1}$ is filtered out but some of the non-joinable ones could survive because of the false positives (*i.e.*, $R_{0}{⋊}_{A=A}R_{1}\subseteq$ $R_{0}{⋊}_{A=A}^{bf}R_{1}\subseteq$ $R_{1}$, where ${⋊}_{A=A}^{bf}$ denotes the semijoin implemented using a Bloom filter). Since bf{$R_{0},\;$*A*} is a bit vector, it is usually much smaller than $R_{0}$[A]. In WSNs, it would be more energy-efficient to send bf{$R_{0},\;$*A*} than to send $R_{0}$[A], provided that the false positives could be properly handled.

When the length of a *k*-transform Bloom filter is *m*, and the number of values in *S* is *n*, the probability of a false positive is approximately:

(1) $${\left(1-{\left(1-\frac{1}{m}\right)}^{kn}\right)}^{k}\approx \;{\left(1-e^{-\frac{kn}{m}}\right)}^{k}$$

and it is minimized to $1/2^{k}$ when k=(m/n)⋅ln2 [16,17]. In this case, the number of bits used to represent a value in *S* is m/n= k/ln2.

### 3.3. SRJA

Given an iceberg join of ${𝓡}_{0}$ and ${𝓡}_{1}$ on attribute *A* in WSNs, SRJA works as follows [6]: The preprocessing as described in Section 2 is carried out to obtain $R_{0}$ and $R_{1}.\;$At ${\tilde{n}}_{0}$ and ${\tilde{n}}_{1}$, the values in $R_{0}\left[\text{A}\right]$ and $R_{1}\left[\text{A}\right]$ is respectively divided into a sequence of *value ranges* with the information on the *count* attribute associated with each range. A range is defined as a 4-tuple (*minval*, *maxval*, *mincount*, *maxcount*). *minval* and *maxval* are respectively the minimum and maximum value of *A* in the range, and the *mincount* and *maxcount* are respectively the minimum and maximum value of *count* among all the counts associated with the values of *A* in the range. The sequence of these ranges constitutes the synopsis of the values of *A* and *count* in $R_{i}$ (*i* = 0, 1). A value range represented as an interval would be much smaller than the list of all the values in the range. In WSNs, it is a common practice to send a value range (*i.e.*, an interval [*minval*, *maxval*]) instead of the full list of values in the range to reduce data transmission cost though accuracy is compromised [18].

**Figure 2:**
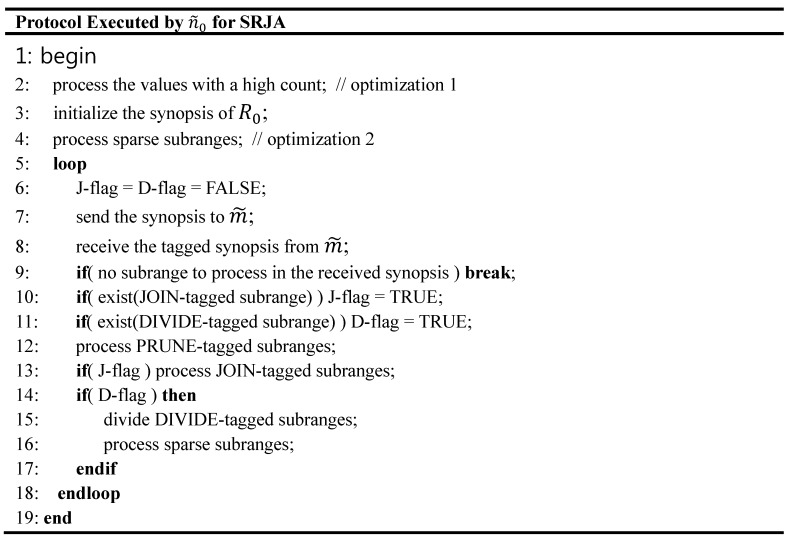
Protocol Executed by ${\tilde{n}}_{0}$ for SRJA.

${\tilde{n}}_{0}$ and ${\tilde{n}}_{1}\;$ send the synopses of $R_{0}$ and $R_{1}$ to the sensor node $\tilde{m}$ located at the midpoint between ${\tilde{n}}_{0}$ and ${\tilde{n}}_{1}$. At $\tilde{m}$, the value range matching is conducted first with the *minval* and *maxval* of the ranges in the synopses. In this matching process, the original synopses are modified. Some ranges of a synopsis are deleted because there is no matched counterpart in the other synopsis or further divided into subranges so that the two synopses have exactly the same set of ranges. Then, for each pair of matched ranges of $R_{0}$ and $R_{1}$, the *mincount*’s and *maxcount*’s are checked, and the ranges are tagged as PRUNE, JOIN, or DIVIDE. The pair for which *maxcount*($R_{0}$) × *maxcount*($R_{1}$) <α is tagged as PRUNE. The pair for which *mincount*($R_{0}$) × *mincount*($R_{1}$) ≥α is tagged as JOIN. The remaining pairs are tagged as DIVIDE. The tagged synopses are sent back to ${\tilde{n}}_{0}$ and ${\tilde{n}}_{1}$. The tuples in a PRUNE range are deleted from $R_{i}$ (*i* = 0, 1). The tuples in a JOIN range not qualified for the query are filtered out by 2-way semijoins, and the qualified ones are finally joined for the result. A DIVIDE range is further divided into subranges. The synopsis of $R_{i}$ is reconstructed with them. This process is repeated until the final query result is obtained. The optimization techniques for SRJA are as follows [6]:

- Optimization 1: Before the above process begins, the tuples whose value of *count* exceeds *α* is sent separately for checking their joinability because they are likely to be qualified for the query.
- Optimization 2: The tuples whose value of *A* belongs to a sparse range are also separately sent. Eliminating a sparse range would make the synopsis more selective.
- Optimization 3: For a range to be tagged as DIVIDE, the *maxcount* of the opposite range (*opp_maxcount*) is also sent when the tagged synopses are sent back. When the range is divided into subranges, the tuples which turn out not to be qualified for the query (*count* ×opp_*maxcount* <α) are deleted.

The skeleton of the protocol executed by ${\tilde{n}}_{0}$ for SRJA is described in Figure 2. The one for ${\tilde{n}}_{1}$ is symmetrical. In [6], it was shown that SRJA significantly outperformed the following baseline schemes:

- NAÏVE: The external join where all the tuples of ${𝓡}_{0}$ and ${𝓡}_{1}$ are sent to the base station.
- SIJ: The synopsis join of [2] extended for iceberg joins where $R_{0}$ and $R_{1}$ are sent to $\tilde{m}$ and fully reduced there.

## 4. Overview of Our Approach

Given an iceberg join of ${𝓡}_{0}$ and ${𝓡}_{1}$ on attribute *A* in WSNs, suppose the preprocessing described in Section 2 has been carried out to obtain $R_{0}$ and $R_{1}.\;$In this section, the main components of our scheme in fully reducing $R_{0}$ and $R_{1}$ are described. They include *2-way fragment semijoin*, *Low Count Cut*, *Highest Count First strategy*, and the *sequence* of 2-way fragment semijoins. The issue of optimization is dealt with in Section 5. The notations used in this section and Section 5 are summarized in Table 1.

**Table 1:** Notations.

Notation	Description
$R_{i}$*,* $R_{1-i}$	$R_{0}$ and $R_{1}$ when *i* = 0; $R_{1}$ and $R_{0}$ when *i* = 1
α	Iceberg threshold
${\tilde{n}}_{i}$	The coordinator sensor node in the region for $R_{i}$ (*i* = 0, 1)
$\tilde{m}\;$	The sensor node located at the midpoint between ${\tilde{n}}_{0}$ and ${\tilde{n}}_{1}$.
*A*	The joining attribute
$D_{A}$	The domain of *A*
||*A*||	The size of a value of *A*
$\bar{⋉}$, $\bar{⋊}$	2-way fragment semijoin operator
$R_{i}^{n}$	A logical fragment of $R_{i}$ defined as ${\text{σ}}_{count\;=\;n}\;R_{i}$ (*i* = 0, 1)
$M_{i}$	The initial maximum value of the attribute count in $R_{i}$ (*i* = 0, 1)
$H_{i}$	The current highest value of the attribute count in $R_{i}$ (*i* = 0, 1)
$L_{i}$	The current lowest value of the attribute count in $R_{i}$ (*i* = 0, 1)
$R_{i}\left(H_{i},H_{1-i}\right)$	The current state of $R_{i}$ after reduced by a sequence of fragment semijoins (*i* = 0, 1)
$R_{i}^{n}\left(H_{i},H_{1-i}\right)$	A logical fragment of $R_{i}\left(H_{i},H_{1-i}\right)$ defined as ${\text{σ}}_{count\;=\;n}\;R_{i}\left(H_{i},H_{1-i}\right)$ (*i* = 0, 1)
${\bar{⋉}}_{i}\left(H_{i},H_{1-i}\right)$	A fragment semijoin for which $R_{i}^{H_{i}}\left(H_{i},H_{1-i}\right)$ is the reducer relation
$S_{\bar{⋉}\text{*}}^{i}\left(H_{i},H_{1-i}\right)$	Given $R_{i}\left(H_{i},H_{1-i}\right)$ and $R_{1-i}\left(H_{1-i},H_{i}\right)$, the optimal sequence of fragment semijoins that fully reduces $R_{0}$ and $R_{1}$ provided that the first semijoin is ${\bar{⋉}}_{i}\left(H_{i},H_{1-i}\right)$ (*i* = 0, 1)
$C_{\bar{⋉}\text{*}}^{i}\left(H_{i},H_{1-i}\right)$	The cost of $S_{\bar{⋉}\text{*}}^{i}\left(H_{i},H_{1-i}\right)$
${\hat{c}}_{\bar{⋉}}^{i}\left(H_{i},H_{1-i}\right)$	The cost of ${\bar{⋉}}_{i}\left(H_{i},H_{1-i}\right)$
$n_{\bar{⋉}\text{*}}^{i}\left(H_{i},H_{1-i}\right)$	The value of attribute count in $R_{i}$ with which (Equation (2)) is minimized (*i* = 0, 1). Let *n* = $n_{\bar{⋉}\text{*}}^{i}\left(H_{i},H_{1-i}\right)$. Then, the following sequence of fragment semijoins is the prefix of $S_{\bar{⋉}\text{*}}^{i}\left(H_{i},H_{1-i}\right)$: ${\bar{⋉}}_{i}\left(H_{i},H_{1-i}\right)\cdot {\bar{⋉}}_{i}\left(H_{i}-1,H_{1-i}\right)\cdot {\bar{⋉}}_{i}\left(H_{i}-2,H_{1-i}\right)\cdot \cdot \cdot {\bar{⋉}}_{i}\left(n,H_{1-i}\right)$.
$BF_{i}\left(H_{i},H_{1-i}\right)$	The Bloom filter sent for ${\bar{⋉}}_{i}\left(H_{i},H_{1-i}\right)$
$\left||BF_{i}\left(H_{i},H_{1-i}\right)|\right|$	The size of $BF_{i}\left(H_{i},H_{1-i}\right)$
$k_{i}\;\left(H_{i},H_{1-i}\right)$	The optimal number of hash functions used for $BF_{i}\left(H_{i},H_{1-i}\right)$
$C_{fp}^{i}\left(H_{i},H_{1-i}\right)$	The cost of handling false positives with $BF_{i}\left(H_{i},H_{1-i}\right)$ in executing ${\bar{⋉}}_{i}\left(H_{i},H_{1-i}\right)$

## 4.1. 2-Way Fragment Semijoin

As described in Section 2, $R_{0}$ and $R_{1}\;$are the base histograms of ${𝓡}_{0}$ and ${𝓡}_{1}$ with attribute *count*. For relation $R_{i}$ (*i* = 0, 1), let $R_{i}^{n}$ denote the result of ${\text{σ}}_{count\;=\;n}\;R_{i}$. $R_{i}^{n}$ is a horizontal *fragment* of $R_{i}$ on count, and thus, a horizontal subset of the base histogram of ${𝓡}_{i}$. For example, given an iceberg join between ${𝓡}_{0}$ and ${𝓡}_{1}$ in Figure 1 on attribute A, $R_{0}=\text{\{}\left(\text{A},\text{ count}\right)\;\text{|}\left(1,1\right),\;\left(2,1\right),\;\left(4,2\right)\}$, and $R_{1}=\text{\{}\left(\text{A},\text{ count}\right)\;\text{|}\left(1,2\right),\;\left(2,1\right),\;\left(3,1\right)\}$. Thus, $R_{0}^{1}=\left\{\left(1,1\right),\;\left(2,1\right)\right\}$, $R_{0}^{2}=\left\{\left(4,2\right)\right\}$, $R_{1}^{1}=\left\{\left(2,1\right),\;\left(3,1\right)\right\}$, $R_{1}^{2}=\left\{\left(1,2\right)\right\}$.

Let us consider a *logical* fragmentation of $R_{0}$ and $R_{1}\;$on count. Suppose the lowest and highest value of count in $R_{0}$ is 1 and 7, respectively. Then, $R_{0}$ is logically partitioned into 7 fragments: $R_{0}^{1}$, $R_{0}^{2}$, …, $R_{0}^{7}$. Similarly, suppose $R_{1}$ is logically fragmented into $R_{1}^{1}$, …, $\;R_{1}^{8}$. Now let us consider an iceberg join with α= 30. Since $R_{0}$ and $R_{1}$ are fragmented, the semijoin where a fragment is the reducer relation can be used. For example, semijoin $R_{0}^{7}$ ${⋊}_{A=A}$ $R_{1}$ is executed in the following way: $R_{0}^{7}\left[\text{A}\right]$ is sent to $R_{1}$. Since ⌈α/7⌉=5, only those tuples of $R_{1}$ that belong to the fragments $R_{1}^{j}$ where 5≤j≤ 8 could be joinable with the tuples in $R_{0}^{7}$. Thus, only $\cup_{j=5}^{8}R_{1}^{j}$ could be considered as the reduced relation. The tuples in those fragments not joinable with $R_{0}^{7}\left[\text{A}\right]$ are deleted.

Now let us define a new type of operation called *fragment semijoin* by modifying the semijoin. In the semijoin, the joinable tuples of the reduced relation remain whereas the non-joinable ones are deleted. In our fragment semijoin, the tuple filtering is done in the other way around with a side-effect. The joinable ones are deleted whereas the non-joinable ones remain. In other words, the result of a fragment semijoin is the complement of the conventional semijoin (in some commercial DBMSs, this variation of the semijoin is called an *anti join*; in fact, the term *fragment anti join* might be more exact one than fragment semijoin, however, we keep the term semijoin with a modifier “fragment” because it is widely known). Let us denote a fragment semijoin operator as $\bar{⋊}$. With α= 30, a fragment semijoin $R_{0}^{7}$ ${\bar{⋊}}_{A=A}$ $R_{1}$ is executed in the following way: $R_{0}^{7}\left[\text{A}\right]$ is sent to $R_{1}$. The tuples in $\cup_{j=5}^{8}R_{1}^{j}$ not joinable with $R_{0}^{7}\left[\text{A}\right]$ are intact (*i.e*., not deleted). Instead, the joinable ones are deleted and inserted to a separate relation $R\prime_{1}.$ The reason why the unmatched tuples remain is that they might be joined with the tuples in other fragments of $R_{0}$. The insertion of matched tuples to $R\prime_{1}$ is a necessary side-effect of a fragment semijoin. Initially, $R\prime_{1}$ is empty. Every time a fragment semijoin to reduce $R_{1}$ is executed, the matched tuples, if any, are inserted to $R\prime_{1}$. When *all* the tuples of $R_{1}$ that are joinable with $R_{0}$ have been inserted to $R\prime_{1}$, $R_{1}\;$is said to be fully reduced to $R\prime_{1}$. Until then, $R_{1}\;$is said to be reduced to $R\prime \prime_{1}$, which keeps the tuples of $R_{1}$ yet to be checked for joinability with other fragments of $R_{0}$. Note that $R\prime_{1}\cup^{\text{​}}R\prime \prime_{1}\subseteq R_{1}$. The difference, $R_{1}-\left(R\prime_{1}\cup^{\text{​}}R\prime \prime_{1}\right)$, is the set of tuples of $R_{1}$ that have been finally confirmed not joinable with $R_{0}$. How this difference is computed will be explained in the next two subsections. In a *2-way* fragment semijoin, the backward reduction phase is added where the matched joining attribute values are sent back.

So far, we have assumed that the joining attribute values are sent in the forward reduction phase. In our scheme, we employ the Bloom filter as a synopsis of the joining attribute values in executing a fragment semijoin to reduce the amount of data transmission in WSNs. The fragment semijoin with a Bloom filter is the same as above except (1) the Bloom filter constructed from the joining attribute values are sent; and (2) the false positives need to be handled. With α= 30, the fragment semijoin $R_{0}^{7}$ ${\bar{⋊}}_{A=A}$ $R_{1}$ using a Bloom filter is executed in the following way: The values in $R_{0}^{7}.\text{A}$ are represented in a Bloom filter, bf {$R_{0}^{7},\;$*A*}, which is sent to $R_{1}$. For each value of *A* in $\cup_{j=5}^{8}R_{1}^{j}$, the membership test is done with bf {$R_{0}^{7},\;$*A*}. The matched tuples (including those due to false positives) are moved to $R\prime_{1}$. Their values of *A* are sent back to $R_{0}$. The ones that turn out to have been sent due to false positives are sent back to $R_{1}$, and their corresponding tuples are moved from $R\prime_{1}$ back to their original fragments. Note that the backward reduction phase is mandatory with the fragment semijoin using a Bloom filter to sort out false positives. In the rest of this paper, what we mean by a fragment semijoin denoted with $\bar{⋊}\;$ is a 2-way fragment semijoin using a Bloom filter unless stated otherwise.

## 4.2. Low Count Cut (LCC)

In our approach, the cardinality constraint is the primary condition to check. One of the advantages we gain by checking the cardinality constraint first is that we can delete those tuples of $R_{0}$ and $R_{1}$ with low counts without checking the join predicate. Let $M_{0}$ and $M_{1}$ respectively denote the maximum count in $R_{0}$ and $R_{1}$. Then, the tuples in the fragments $R_{0}^{j}$ ($j\leq \lceil \text{α}/M_{1}\rceil$) and $R_{1}^{j}$ ($j\leq \lceil \text{α}/M_{0}\rceil$) cannot meet the cardinality constraint, and they need not be considered at all. For example, suppose $R_{0}$ is fragmented into $R_{0}^{1}$,…,$\;R_{0}^{7}$ whereas $R_{1}$ is fragmented into $R_{1}^{1}$,…,$\;R_{1}^{10}$, and α = 20. $R_{0}^{1}$ is ignored for the join because $M_{1}\;$= 10 (1 × 10 <α). Similarly, so is $\cup_{j=1}^{2}R_{1}^{j}$ because $M_{0}=\;$7 (7 × 1 <α and 7 × 2 <α). In general, the maximum count of one relation determines the minimum count of the candidate tuples for the join in the other relation. The tuples with the count less than this minimum can be deleted without checking the join predicate. Let us call this reduction effect as *Low Count Cut* (*LCC*). The LCCs in the above example are called *initial* LCCs. The initial LCCs for $R_{0}$ would be possible after ${\tilde{n}}_{0}$ is notified of $M_{1}$ as a part of the optimization process, which will be described in Section 5.5. Other than initial ones, LCC could occur after a fragment semijoin is executed. It will be explained in the next subsection

## 4.3. Highest Count First (HCF)

It would be efficient to take advantage of LCCs in reducing $R_{0}$ and $R_{1}$ with a sequence of fragment semijoins. When a fragment of one relation is to be selected as the reducer relation for a fragment semijoin, it is desirable to select the fragment with the *highest* count in that relation, for it would result in LCC in the reduced relation. For example, suppose $R_{0}$ is fragmented into $R_{0}^{3}$, ⋯,$\;R_{0}^{7}$ whereas $R_{1}$ is fragmented into $R_{1}^{4}$, ⋯,$\;R_{1}^{9}$, and α = 25. Suppose the fragment semijoin $R_{0}{\bar{⋉}}_{A=A}R_{1}^{9}$ is executed, and $R_{0}$ is reduced to $R{\text{″}}_{0}$. In $R_{1}$, $R_{1}^{4}$, ⋯,$\;R_{1}^{8}$ remain. In $R{\text{″}}_{0}$, $R{\text{″}}_{0}^{4}$, ⋯,$\;R{\text{″}}_{0}^{7}$ remain. Note that $R{\text{″}}_{0}$ does not contain $R_{0}^{3}$. It is deleted due to LCC. Note that the LCC due to a fragment semijoin occurs only when the fragment with the highest count is the reducer relation. If $R_{0}{\bar{⋉}}_{A=A}R_{1}^{8}$ is executed to reduce $R_{0}$ to $R\prime \prime_{0}$ with $R_{1}^{9}$ remaining, $R\prime \prime_{0}^{3}$ is still contained in $R\prime \prime_{0}$ because some of the tuples in $R{\text{″}}_{0}^{3}$ might be joinable with those in $R_{1}^{9}$.

**Figure 3:**
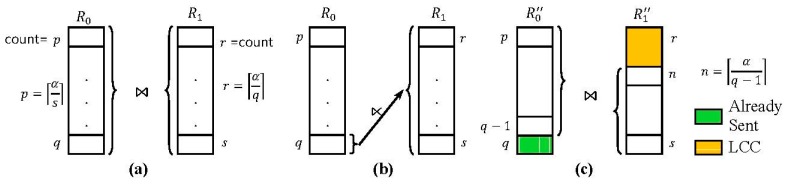
Low Count Cut with Highest Count First Strategy. (**a**) Logical fragmentation of $R_{0}$ and $R_{1}$, where $R_{0}=\cup_{j=p}^{q}R_{0}^{j}$ (*p* ≤ *q*) and$R_{1}=\cup_{j=r}^{s}R_{1}^{j}$ (*r* ≤ *s*); (**b**) fragment semijoin $R_{0}^{q}\;\bar{⋊}\;$$R_{1}$; (**c**) Remaining fragments of $R_{0}$ and $R_{1}$ after the fragment semijoin in (**b**).

Let us call the strategy of selecting the fragment with the highest count as the reducer relation for a fragment semijoin as *Highest Count First* (*HCF*). Figure 3 shows how $R_{0}$ and $R_{1}$ are reduced after a fragment semijoin is executed with the HCF strategy. In Figure 3a, each box with a count value in $R_{0}$ and $R_{1}$ denotes a fragment. For example, the box at the top of $R_{0}$ with count = *p* denotes the fragment $R_{0}^{p}$. Figure 3a shows how $R_{0}$ and $R_{1}$ are logically fragmented. As shown, $R_{0}=\cup_{j=p}^{q}R_{0}^{j}$ (*p* ≤ *q*) and $R_{1}=\cup_{j=r}^{s}R_{1}^{j}$ (*r* ≤ *s*). Figure 3b shows the fragment semijoin $R_{0}^{q}\;\bar{⋊}\;$ $R_{1}$. Figure 3c shows which fragments of $R_{0}$ and $R_{1}$ remain. In $R\prime \prime_{0}$, $R\prime \prime_{0}^{q-1}$ is now the fragment with the highest count. In $R\prime \prime_{1}$, the LCC has occurred, and n=⌈α/(q−1)⌉ is now the lowest count in the remaining fragments.

**Figure 4:**
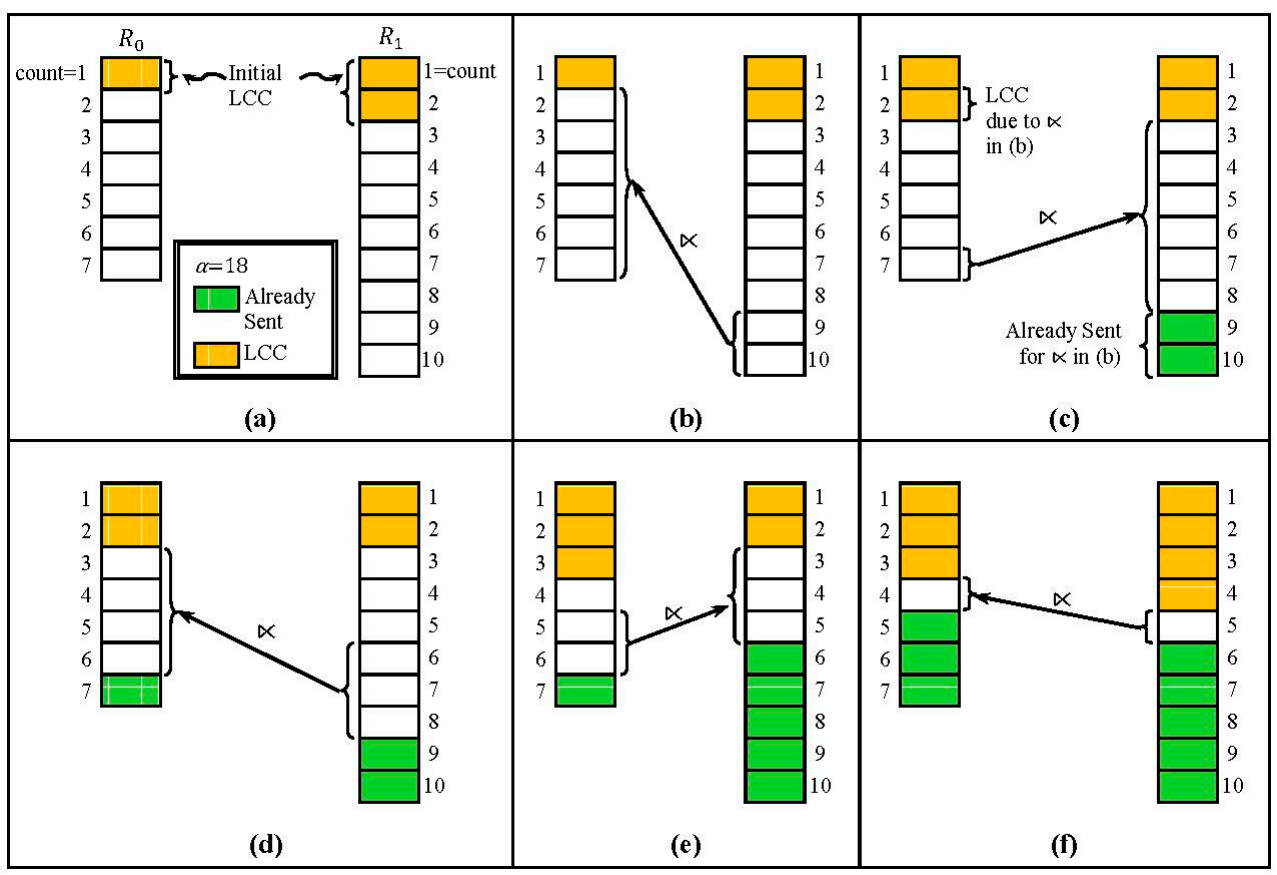
A Sequence of Fragment Semijoins. (**a**) Given $R_{0}=\cup_{j=1}^{7}R_{0}^{j}$ and $R_{1}=\cup_{j=1}^{10}R_{1}^{j}$, the initial LCCs remove the fragments $R_{0}^{1}$, $R_{1}^{1}$, and $R_{1}^{2}$; (**b**–**f**) A sequence of fragment semijoins are executed where the fragments of $R_{0}$ (or $R_{1}$) being the reducer relations, and those of $R_{1}$ (or $R_{0}$) being the reduced relations. After each fragment semijoin, LCCs occur.

### 4.4. Fragment Semijoin Sequence

$R_{0}$ and $R_{1}$ could be fully reduced with a sequence of fragment semijoins which interleaves two types of fragment semijoins: one with a fragment of $R_{0}$ being the reducer relation, and the other with a fragment of $R_{1}$ being the reducer relation. Figure 4 shows an example where $R_{0}=\cup_{j=1}^{7}R_{0}^{j}$ and $R_{1}=\cup_{j=1}^{10}R_{1}^{j}$ are reduced by a sequence of fragment semijoins with α= 18. After the initial LCCs (Figure 4a), a sequence of fragment semijoins are executed after which LCCs occur (Figure 4b–f). Throughout the sequence, either the fragment with the highest count in $R_{0}$ or that in $R_{1}$ is the reducer relation. In Figure 4b,d,e, more than one fragment is depicted as being the reducer relation for fragment semijoins. Since every fragment semijoin selects one fragment at a time as the reducer relation with the HCF strategy, the number of fragment semijoins executed in Figure 4b,d,e, is respectively equal to the number of fragments marked as selected. For example, in Figure 4b, two fragment semijoins are sequentially executed; $R_{0}\;\bar{⋉}$ $R_{1}^{10}$ first, and then $R\prime \prime_{0}\;\bar{⋉}$ $R_{1}^{9}$ where $R\prime \prime_{0}$ is the result of $R_{0}\;\bar{⋉}$ $R_{1}^{10}$.

## 5. Optimal Sequence of Fragment Semijoins

In our approach, a sequence of fragment semijoins are executed to fully reduce $R_{0}$ and $R_{1}$, and the Bloom filter is employed as a synopsis of the joining attribute values. Data transmission for a fragment semijoin occurs to send the Bloom filter and to handle the false positives. According to the Bloom filter theory presented in Section 3.2, the length of a Bloom filter and the number of transformations used could be optimally set to minimize the probability of false positives. In this section, we present an algorithm that generates the optimal sequence of 2-way fragment semijoins with the HCF strategy whereby the total amount of data transmission in fully reducing $R_{0}$ and $R_{1}$ is minimized.

### 5.1. Formulation of Optimization Problem

We develop a *dynamic programming algorithm* to generate the optimal sequence of fragment semijoins that fully reduces $R_{0}$ and $R_{1}$. In describing the algorithm, it is convenient to denote $R_{0}$ and $R_{1}$ as $R_{i}$ and $R_{1-i}$ (*i* = 0, 1). For example, if we need to mention both $R_{0}$ ⋊ $R_{1}$ and $R_{1}$ ⋊ $R_{0}$ to state something that is applied to both (Note that $R_{0}$ ⋊ $R_{1}$ ≠ $R_{1}$ ⋊ $R_{0}$ because the semijoin operation is not commutative.), $R_{i}$ ⋊ $R_{1-i}$ (*i* = 0, 1) will do. In the rest of this paper, the subscripts *i* and 1−i (e.g., $R_{i}$, $R_{1-i}$) are used with (*i* = 0, 1) omitted if they are related to $R_{0}$ or $R_{1}$ and the context is clear. Other notations used in this section are summarized in Table 1.

**Figure 5:**
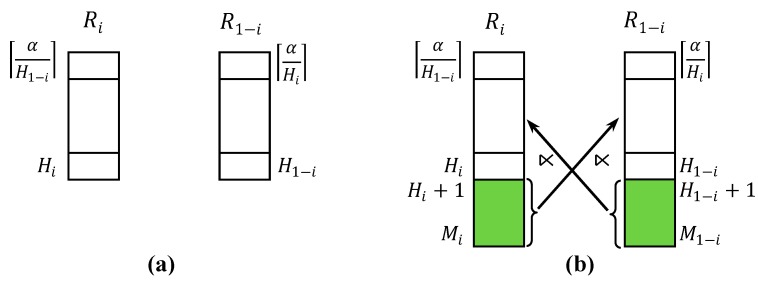
State of $R_{i}$ and $R_{1-i}$ after a certain sequence of fragment semijoins has been executed. (**a**) The current lowest count of $R_{i}$ is ⌈$\text{α}/H_{1-i}$⌉, where $H_{i}$ denotes the current highest count of $R_{i};$; (**b**) Let the initial maximum count of $R_{i}$ be $M_{i}$. Then, each fragment in $\cup_{j=H_{i}+1}^{M_{i}}R_{i}^{j}$ has been selected as the reducer relation for the fragment semijoins executed thus far before or after reduced by some fragments of $R_{1-i}$.

The current state of $R_{i}$ after a certain sequence of fragment semijoins has been executed can be represented with the two current highest counts in the remaining fragments of $R_{i}$ and $R_{1-i}$. Let $H_{i}$ denote the current highest count of $R_{i}$ as depicted in Figure 5. The following two statements hold:

- The current lowest count of $R_{i}$ is $\text{α}/H_{1-i}$ (Figure 5a).
- If the initial maximum count of $R_{i}$ is $M_{i}$, each fragment in $\cup_{j=H_{i}+1}^{M_{i}}R_{i}^{j}$ has been selected as the reducer relation for the fragment semijoins executed thus far before or after reduced by some fragments of $R_{1-i}$ (Figure 5b).

Let $R_{i}\left(H_{i},H_{1-i}\right)$ denote $R_{i}$ reduced thus far, and $R_{i}^{n}\left(H_{i},H_{1-i}\right)$ denote the fragment of $R_{i}\left(H_{i},H_{1-i}\right)$ defined by ${\text{σ}}_{count\;=\;n}\;R_{i}\left(H_{i},H_{1-i}\right)$. Given $R_{i}\left(H_{i},H_{1-i}\right)$ and $R_{1-i}\left(H_{1-i},\;H_{i}\right)$, let ${\bar{⋉}}_{i}\left(H_{i},H_{1-i}\right)$ denote the fragment semijoin $R_{i}^{H_{i}}\left(H_{i},H_{1-i}\right)\;\bar{⋊}$ $R_{1-i}\left(H_{1-i},\;H_{i}\right)$, and $S_{\bar{⋉}\text{*}}^{i}\left(H_{i},H_{1-i}\right)$ denote the optimal sequence of fragment semijoins provided that the first one is ${\bar{⋉}}_{i}\left(H_{i},H_{1-i}\right).$ Let $C_{\bar{⋉}\text{*}}^{i}\left(H_{i},H_{1-i}\right)$ denote the cost of $S_{\bar{⋉}\text{*}}^{i}\left(H_{i},H_{1-i}\right)$, where the cost is defined to be the total amount of data transmission in bits. $C_{\bar{⋉}\text{*}}^{i}\left(H_{i},H_{1-i}\right)$ is given in the following *recurrence relation*:

(2) $$C_{\bar{⋉}\text{*}}^{i}\left(H_{i},H_{1-i}\right)=\underset{|\frac{\text{α}}{H_{1-i}}|\;\leq \;n\;\leq H_{i}}{\min}\left\{\;\overset{n}{\underset{j=H_{i}}{\sum }}{\hat{c}}_{\bar{⋉}}^{i}\left(j,H_{1-i}\right)+C_{\bar{⋉}\text{*}}^{1-i}\left(H_{1-i},n-1\right)\;\right\},i=0,\;1$$

${\hat{c}}_{\bar{⋉}}^{i}\left(j,H_{1-i}\right)$ denotes the cost of ${\bar{⋉}}_{i}\left(j,H_{1-i}\right)$. Thus, the term $\overset{n}{\underset{j=H_{i}}{\sum }}{\hat{c}}_{\bar{⋉}}^{i}\left(j,H_{1-i}\right)$ is the total cost of executing the following sequence of fragment semijoins: ${\bar{⋉}}_{i}\left(H_{i},H_{1-i}\right)\cdot {\bar{⋉}}_{i}\left(H_{i}-1,H_{1-i}\right)\cdot {\bar{⋉}}_{i}\left(H_{i}-2,H_{1-i}\right)\cdot \cdot \cdot {\bar{⋉}}_{i}\left(n,H_{1-i}\right)$. With this sequence, the fragments $R_{i}^{j}\left(H_{i},H_{1-i}\right)$ where $j=H_{i}$, $H_{i}-1,\;H_{i}-2,\;\cdots ,\;n$ are to be selected as the reducer relations in the descending order of *j* according to the HCF strategy.

The *termination condition* of this recurrence relation is ⌈$\text{α}/H_{1-i}\rceil <H_{i}\;\text{or}\;\lceil \text{α}/H_{i}\rceil <H_{1-i}$, which means either one of the two relations gets empty. In such a case, no further fragment semijoin is needed. Thus:

(3) $$C_{\bar{⋉}\text{*}}^{i}\left(H_{i},H_{1-i}\right)=0\text{ if }\lceil \frac{\text{α}}{H_{1-i}}\rceil <H_{i}\;\text{or}\;\lceil \frac{\text{α}}{H_{i}}\rceil <H_{1-i}$$

Let $n_{\bar{⋉}\text{*}}^{i}\left(H_{i},H_{1-i}\right)$ denote the value *n* for which $C_{\bar{⋉}\text{*}}^{i}\left(H_{i},H_{1-i}\right)$ in (Equation (2)) is minimized. That is,

(4) $$n_{⋉\text{*}}^{i}\left(H_{i},H_{1-i}\right)=\underset{|\frac{\text{α}}{H_{1-i}}|\;\leq \;n\;\leq \;H_{i}}{minarg}\left\{\;\overset{n}{\underset{j=H_{i}}{\sum }}{\hat{c}}_{\bar{⋉}}^{i}\left(j,H_{1-i}\right)+C_{\bar{⋉}\text{*}}^{1-i}\left(H_{1-i},n-1\right)\;\right\},\;i=0,\;1$$

Let $M_{i}$ be the maximum count in $R_{i}$. Then, the optimal sequence of fragment semijoins for $R_{0}$ and $R_{1}$ is either $S_{\bar{⋉}\text{*}}^{0}\left(M_{0},M_{1}\right)$ or $S_{\bar{⋉}\text{*}}^{1}\left(M_{1},M_{0}\right)$. Its cost is $\min\left\{C_{\bar{⋉}\text{*}}^{0}\left(M_{0},M_{1}\right),C_{\bar{⋉}\text{*}}^{1}\left(M_{1},M_{0}\right)\right\}\;$or $\underset{i\in \left\{0,1\right\}}{\min}\left\{C_{\bar{⋉}\text{*}}^{i}\left(M_{i},M_{1-i}\right)\right\}$. Let $j=\underset{i\in \left\{0,1\right\}}{minarg}\left\{C_{\bar{⋉}\text{*}}^{i}\left(M_{i},M_{1-i}\right)\right\}$. The value of *j*, which is 0 or 1, indicates that the first fragment semijoin in the optimal sequence is ${\bar{⋉}}_{j}\left(M_{j},M_{1-j}\right)$ where $R_{j}^{M_{j}}$ is the reducer relation. Thus, the optimal sequence can be represented as *i* (i∈{0,1}) followed by a sequence of counts in $R_{i}$ and $R_{1-i}$ as follows:

$$i;n_{i}^{1},\;n_{1-i}^{1},\;n_{i}^{2},\;n_{1-i}^{2},\;\cdots ,\;n_{i}^{k},\;n_{1-i}^{k},\;\cdots ,\;$$

where

$$n_{i}^{1}=n_{\bar{⋉}*}^{i}\left(M_{i},\;M_{1-i}\right)$$

$$n_{1-i}^{1}=n_{\bar{⋉}*}^{1-i}\left(M_{1-i},\;n_{i}^{1}-1\right)$$

$$n_{i}^{2}=n_{\bar{⋉}*}^{i}\left(n_{i}^{1}-1,\;n_{1-i}^{1}-1\right)$$

$$n_{1-i}^{2}=n_{\bar{⋉}*}^{1-i}\left(n_{1-i}^{1}-1,\;n_{i}^{2}-1\right)$$

For example, if the sequence of fragment semijoins in Figure 4 is the optimal one, then it is represented as 1, 9, 7, 6, 5, 5.

### 5.2. Cost of 2-Way Fragment Semijoin

The cost of a fragment semijoin ${\hat{c}}_{\bar{⋉}}^{i}\left(x,y\right)$ where *i* = 0, 1 is defined as the total amount of data transmission in bits, and given as follows:

(5) $${\hat{c}}_{\bar{⋉}}^{i}\left(x,y\right)=||BF_{i}\left(x,y\right)||+C_{fp}^{i}\left(x,y\right)$$

The first term denotes the size of the Bloom filter, and the second is for the cost of handling false positives. Since the total cost of sending all the joinable attribute values (excluding those from false positives) in the backward reduction phases is the same for all the possible sequences of fragment semijoins, it is omitted. The multiplication of the number of hops between the two regions of $R_{0}$ and $R_{1}$ is also omitted because it is the same for all the sequences.

#### 5.2.1. Size of Bloom Filter

||$BF_{i}\left(x,y\right)$|| denotes the size of the Bloom filter $BF_{i}\left(x,y\right)$, which represents the set of joining attribute values in $R_{i}^{x}\left(x,y\right)$ to be sent in executing ${\bar{⋉}}_{i}\left(x,y\right)$. When this fragment semijoin is to be executed, the reduced state of $R_{i}$ and $R_{1-i}$ are respectively $R_{i}\left(x,y\right)$ and $R_{1-i}\left(y,x\right)$. That is, $H_{i}\;$= *x*, $H_{1-i}\;$= *y*, and $BF_{i}\left(x,y\right)$ is to represent the set of joining attribute values in $R_{i}^{x}\left(x,y\right)$. The joining attribute values in $R_{i}\;$ are unique among all the tuples. Thus, the number of distinct values in the joining attribute *A* of $R_{i}^{x}\left(x,y\right)$ is $\left|R_{i}^{x}\left(x,y\right)\right|$. This number can be estimated as follows using the semijoin selectivity estimation described in Section 3.1:

(6) $$\left|R_{i}^{x}\left(x,y\right)\right|=\left|R_{i}^{x}\right|\cdot \left(1-\frac{\left|\cup_{j=y+1}^{M_{1-i}}R_{1-i}^{j}\right|}{\left|D_{A}\right|}\right)$$

$R_{i}$ and $R_{1-i}$ have been reduced by each other with a fragment semijoin sequence until ($H_{i},H_{1-i}$) becomes (*x, y*). However, the above estimation is valid since the joining attribute values are unique in $R_{i}$, that is, $R_{i}^{p}\cap^{\text{​}}R_{i}^{q}$ = ∅ (*p* ≠ *q*), and thus, the following holds: $R_{i}^{p}$ $⋉R_{1-i}=$ $R_{i}^{p}$ ⋉ $R_{1-i}$ $⋉\;R_{i}^{q}$) where *p* ≠ *q*.

Now $\left||BF_{i}\left(x,y\right)|\right|$. can be estimated as follows according to the Bloom filter theory described in Section 3.2:

(7) $$||BF_{i}\left(x,y\right)||=\frac{\left|R_{i}^{x}\left(x,y\right)\right|\cdot k_{i}\left(x,y\right)}{\ln2}$$

where $k_{i}\left(x,y\right)$ denotes the *optimal* number of hash functions for $BF_{i}\left(x,y\right)$, which will be explained shortly. In some cases, the number of tuples of $R_{i}^{x}\left(x,y\right)$ is so small that the size of the list of joining attribute values might be smaller than $\left||BF_{i}\left(x,y\right)|\right|$. In such a case, the list is sent instead of $BF_{i}\left(x,y\right).$ To cover such exceptions, we modify the above equation as follows:

(8) $$||BF_{i}\left(x,y\right)||=min\left\{\;\frac{\left|R_{i}^{x}\left(x,y\right)\right|\cdot k_{i}\left(x,y\right)}{\ln2},\;\left|R_{i}^{x}\left(x,y\right)\right|\cdot ||A||\;\right\}+1$$

where ||A|| denotes the number of bits to represent a value in the joining attribute *A*, and the one added is for a flag bit (for distinguishing a Bloom filter from a value list).

#### 5.2.2. Cost of Handling False Positives

The cost of handling the false positives is estimated as follows: Let *D*, *E*, and *F* respectively denote the number of joining attribute values in $R_{1-i}$ for which the membership tests with $BF_{i}\left(x,y\right)$ are to be done, the number of non-joinable tuples among those *D* values, and the number of false positives out of those *E* values. Then:

(9) $$D=\left|\cup_{j=|\frac{\text{α}}{x}|}^{y}R_{1-i}^{j}\left(y,x\right)\right|,\;E=D\cdot \left(1-\frac{\left|{\text{R}}_{i}^{x}\left(x,y\right)\right|}{\left|D_{A}\right|}\right),\;\text{and}\;F=E\cdot \frac{1}{2^{k_{i}\left(x,y\right)}}$$

The estimation of *E* is according to the semijoin selectivity estimation as described in Section 3.1. In the equation of *F*, $1/2^{k_{i}\;\left(x,y\right)}$ is the probability of a false positive as summarized in Section 3.2. The values of the false positives are sent back to $R_{i}$ in the backward reduction phase, and then sent back to $R_{1-i}$ after the false positives are confirmed. Thus, the cost of handling the false positives for a fragment semijoin is 2 ⋅ *F* ⋅||A||*.* That is:

(10) $$C_{fp}^{i}\left(x,y\right)=\frac{1}{2^{k_{i}\;\left(x,y\right)-1}}\cdot \left|\cup_{j=|\frac{\text{α}}{x}|}^{y}R_{1-i}^{j}\left(y,x\right)\right|\cdot \left(1-\frac{\left|{\text{R}}_{i}^{x}\left(x,y\right)\right|}{\left|D_{A}\right|}\right)\cdot ||A||$$

In case that the list of joining attribute values is sent instead of $BF_{i}\left(x,y\right)$, $C_{fp}^{i}\left(x,y\right)=\;$0.

#### 5.2.3. Optimal Number of Hash Functions.

$k_{i}\left(x,y\right)$, the optimal number of hash functions for $BF_{i}\left(x,y\right)$, is determined as follows: as summarized in Section 3.2, the probability of false positives is affected by the number of hash functions. As the number of hash functions increases, the length of the Bloom filter increases with the probability of false positives decreased. In (Equation (5)), the cost of a fragment semijoin, ${\hat{c}}_{\bar{⋉}}^{i}\left(x,y\right)$, is given as a sum of two terms, $\left||BF_{i}\left(x,y\right)|\right|$ and $C_{fp}^{i}\left(x,y\right)$. There exists a tradeoff between the two terms, and thus, $k_{i}\left(x,y\right)$ should be determined to be a positive integer such that ${\hat{c}}_{\bar{⋉}}^{i}\left(x,y\right)$ is minimized. From Equations (8) and (10), the cost of a fragment semijoin ${\bar{⋉}}_{i}\left(x,y\right)$ with *k* hash functions is given as follows:

(11) $$\frac{\left|R_{i}^{x}\left(x,y\right)\right|\cdot k}{\ln2}+\frac{1}{2^{k-1}}\cdot \left|\cup_{j=|\frac{\text{α}}{x}|}^{y}R_{1-i}^{j}\left(y,x\right)\right|\cdot \left(1-\frac{\left|{\text{R}}_{i}^{x}\left(x,y\right)\right|}{\left|D_{A}\right|}\right)\cdot ||A||+1$$

This equation can be given as a function of k: $f\left(k\right)=\text{a}\cdot k+b/2^{k}+\;c$, where *a*, *b*, and *c* are constants. The differentiation of f(k) with respect to k reveals that $k_{i}\left(x,y\right)=\lceil \log_{2}\left(\left(b/a\right)\cdot \ln2\right)\rceil$, where *a*$\;=\left|R_{i}^{x}\left(x,y\right)\right|/\ln2$, *b*$\;=2\cdot \left|\cup_{j=\text{α}/x}^{y}R_{1-i}^{j}\left(y,x\right)\right|\cdot \left(1-\left(\left|R_{i}^{x}\left(x,y\right)\right|/\left|D_{A}\right|\right)\right)\cdot ||A||$. If this value is less than 1, $k_{i}\left(x,y\right)$ is set to 1.

### 5.3. Dynamic Programming Algorithm

**Figure 6:**
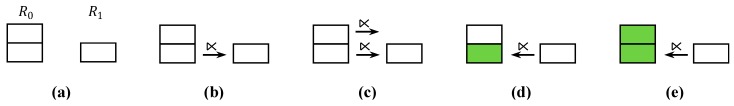
Optimal Solution from Initialization. (**a**) Two fragments remaining in $R_{0}$ and one in $R_{1}$. There are only two possible cases for $S_{\bar{⋉}\text{*}}^{0}\left(H_{0},H_{1}\right)$, (**b**) One case, (**c**) The other case, (**d**) The optimal solution (*i.e*., $S_{\bar{⋉}\text{*}}^{1}\left(H_{1},H_{0}\right)$) for the case of (b) is given from the initialization, (**e**) The same for the case of (**c**).

**Figure 7:**
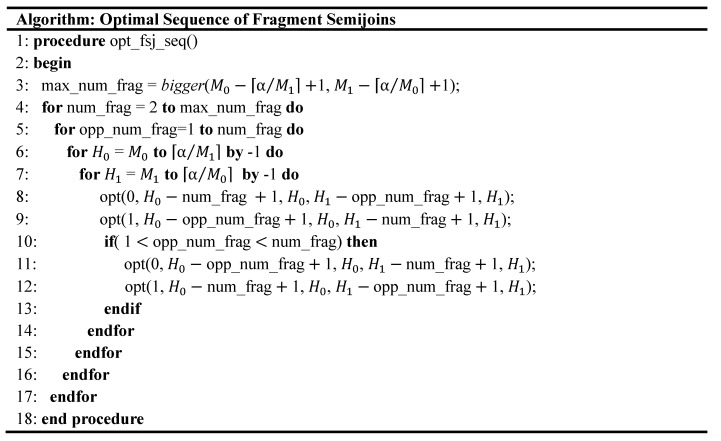
Algorithm for Generating Optimal Sequence of Fragment Semijoins.

$C_{\bar{⋉}\text{*}}^{\text{i}}$ (x,y) and $n_{⋉\text{*}}^{i}$ (x,y) can be obtained with a dynamic programming algorithm. From (Equation (2)) through (Equation (4)), $C_{\bar{⋉}\text{*}}^{\text{i}}$ (x,y) and $n_{⋉\text{*}}^{i}$ (x,y) are initialized as follows: If there is no fragment remaining in either one of $R_{0}$ and $R_{1}$, that is, if x<⌈α/y⌉ or y<⌈α/x⌉, then $C_{\bar{⋉}\text{*}}^{i}$ (x,y) is set to 0, and $n_{⋉\text{*}}^{i}$ (x,y) is undefined. If none of $R_{0}$ and $R_{1}$ is empty but there remains only one fragment in $R_{i}$ (*i.e.*, x=⌈α/y⌉), the only possible fragment semijoin is the one where that fragment is the reducer relation. Thus, $C_{\bar{⋉}\text{*}}^{i}$(x,y)$\;={\hat{c}}_{\bar{⋉}}^{i}\left(x,y\right)$ and $n_{⋉\text{*}}^{i}$ (x,y) =x. Starting from these initializations, the optimal solution for the cases where the number of remaining fragments of $R_{0}$ and $R_{1}$ is greater than 1 can be obtained. For example, suppose there are two fragments remaining in $R_{0}$ and one in $R_{1}$ (Figure 6a). $S_{\bar{⋉}\text{*}}^{0}\left(H_{0},H_{1}\right)$ can be obtained as follows: Because of the HCF strategy, the fragment with the highest count is the reducer relation in the first semijoin (Figure 6b). The remaining fragment of $R_{0}$ might be the reducer relation of the next semijoin (Figure 6c). That is, Figure 6b,c show all the possible cases. Figure 6d,e respectively show the subsequent fragment semijoin where the fragment of $R_{1}$ is the reducer relation after the semijoins in Figure 6b,c assuming that no LCC has occurred. The optimal solutions (*i.e*., $S_{\bar{⋉}\text{*}}^{1}\left(H_{1},H_{0}\right))\;$in Figure 6d,e are already known, because the number of remaining fragment in $R_{0}$ is 1 (Figure 6d) or 0 (Figure 6e), and that in $R_{1}$ is 1. The optimal solutions here are already given from the initialization. In this way, $S_{\bar{⋉}\text{*}}^{i}\left(H_{i},H_{1-i}\right)$ can be obtained when there are 3, 4,…, $M_{i}-\lceil \text{α}/M_{1-i}\rceil +\;1\;$fragments remaining in $R_{i}$ (*i* = 0, 1). Figure 7 describes the algorithm that carries out this process.

**Figure 8:**
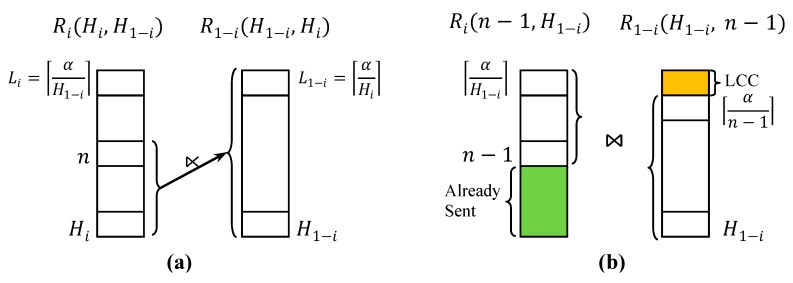
Optimal Solution Obtained with Procedure *opt*(*i*, $L_{0}$, $H_{0}$, $L_{1}$, $H_{1}$), where $L_{i}$ and $H_{i}$ are respectively the current lowest and highest count in $R_{i}$. (**a**) The optimal value of *n* for $S_{\bar{⋉}\text{*}}^{i}\left(H_{i},H_{1-i}\right)$ is obtained; (**b**) using the optimal solution, $S_{\bar{⋉}\text{*}}^{1-i}\left(H_{1-i},n-1\right)$, for each value of *n*.

In the algorithm, procedure *opt*(*i,* $L_{0}$, $H_{0}$, $L_{1}$, $H_{1}$) is invoked. As shown in Figure 8a, the arguments $L_{0}$ and $H_{0}$ are respectively the current lowest and highest count in $R_{0}$. $L_{1}$ and $H_{1}$ are those in $R_{1}$. Procedure *opt*(*i*, $L_{0}$;, $H_{0}$, $L_{1}$, $H_{1}$) vacuously returns unless $L_{0}=\lceil \text{α}/H_{1}\rceil$ and $L_{1}=\lceil \text{α}/H_{0}\rceil$. If the arguments are valid ones, it finds $S_{\bar{⋉}\text{*}}^{i}\left(H_{i},H_{1-i}\right)$. When *i* = 0, opt(0, $L_{0}$, $H_{0}$, $L_{1}$, $H_{1}$) finds out a value *n* in the range [$L_{0}$, $H_{0}$] that minimizes $\overset{n}{\underset{j=H_{0}}{\sum }}{\hat{c}}_{\bar{⋉}}^{0}\left(j,H_{1}\right)+C_{\bar{⋉}\text{*}}^{1}\left(H_{1},n-1\right)$. (Figure 8a corresponds to the first term, and Figure 8b to the second one.) In doing so, $C_{\bar{⋉}\text{*}}^{0}$($H_{0}$, $H_{1}$) and $n_{⋉\text{*}}^{0}$($H_{0}$, $H_{1}$) are obtained. Similarly, when *i* = 1, opt(1, $L_{0}$, $H_{0}$, $L_{1}$, $H_{1}$) finds out a value *n* in the range [$L_{1}$, $H_{1}$] that minimizes $\overset{n}{\underset{j=H_{1}}{\sum }}{\hat{c}}_{\bar{⋉}}^{1}\left(j,H_{0}\right)+C_{\bar{⋉}\text{*}}^{0}\left(H_{0},n-1\right)$. $C_{\bar{⋉}\text{*}}^{1}$($H_{1}$, $H_{0}$) and $n_{⋉\text{*}}^{1}$($H_{1}$, $H_{0}$) are obtained.

The variable *num_frag* denotes the number of remaining fragments in $R_{i}$, and *opp_num_frag* denotes that in $R_{1-i}$. In the two outermost loops, the values of these variables increase. In the two innermost loops, pairings of $H_{0}$ and $H_{1}$ are provided such that the difference between $H_{i}$ and $L_{i}$ is set by *num_frag* and *opp_num_frag, i =* 0, 1. The invocations of *opt*() in Line 8 and 9 find the optimal solution for the case where the number of fragments in the reducer relation is greater than that in the reduced relation. Those in Line 11 and 12 find the optimal solution for the inverse case.

**Figure 9:**
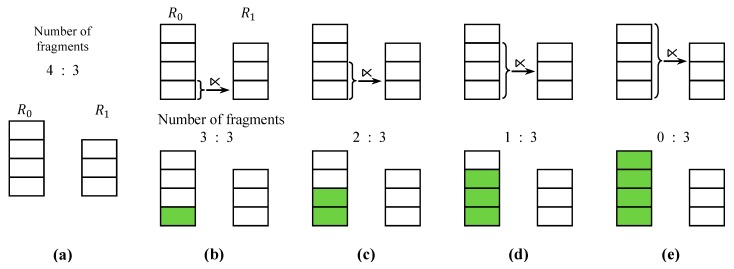
An Example of Optimization with Procedure *opt*(). (**a**) 4 and 3 fragments remaining in $R_{0}$ and $R_{1}$, respectively, (**b**–**e**) For the remaining fragments in (**a**), 4 cases that could result in the optimal solution, $S_{\bar{⋉}\text{*}}^{0}\left(H_{0},H_{1}\right)$, are shown at the top. Determining which case leads to the optimal solution is possible, because the optimal solution, $S_{\bar{⋉}\text{*}}^{1}\left(H_{1},H_{0}\right)$, for the remaining fragments in each of the bottom of (**b**–**e**) is already known.

For example, suppose four and three fragments remain in $R_{0}$ and $R_{1}$, respectively (Figure 9a). Let us consider $S_{\bar{⋉}\text{*}}^{0}\left(H_{0},H_{1}\right)$ for them. Figure 9b–e show that there could be four cases possible with the HCF strategy. After one,  ⋯, four fragments of $R_{0}$ are used as the reducer relations in succession, the remaining fragments are shown at the bottom of Figure 9b–e, respectively. To find out which case of the four would lead to $S_{\bar{⋉}\text{*}}^{0}\left(H_{0},H_{1}\right)$, $S_{\bar{⋉}\text{*}}^{1}\left(H_{1},H_{0}\right)$ for the fragments in each of the bottom of Figure 9b–e should be already known (see (Equation (2))). In other words, before $S_{\bar{⋉}\text{*}}^{0}\left(H_{0},H_{1}\right)$ for the fragments in Figure 9a is obtained, $S_{\bar{⋉}\text{*}}^{1}\left(H_{1},H_{0}\right)$ for the fragments in each of the bottom of Figure 9b–e should have been obtained. The algorithm in Figure 7 guarantees this by calling procedure *opt*() in proper order through the nested loops.

The complexity of the algorithm inFigure 7 is $\text{Ο}\left(f_{0}\cdot f_{1}\cdot \left(\frac{f\left(f+1\right)}{2}-1\right)\right)$ where $f_{i}=M_{\text{i}}-\lceil \alpha /M_{1-\text{i}}\rceil +1$, which is the number of fragments in $R_{i}$ remaining after initial LCCs (*i* = 0, 1), and *f* = *max*($f_{0}$, $f_{1}$). The innermost two for loops (line 6 and 7 in Figure 7) combined are repeated $f_{0}\cdot f_{1}$ times. The second outermost for loop (line 5) is repeated *i* times when num_frag = *i* at the outermost for loop. Since num_frag at the outermost for loop varies from 2 to *f*, the computation in the nested for loops (lines 8–13) would be repeated $f_{0}\cdot f_{1}\cdot \left(2+\ldots +f\right)$ times. The complexity would be $\text{Ο}\left(f^{4}\right)$ when $f_{0}=f_{1}=f$. Due to the characteristics of iceberg queries which are supposed to produce very selective result, $M_{i}$’s and α would grow or shrink together. Otherwise, the query would not be so selective. It means that the number of fragments remaining after initial LCCs would not be high. When $M_{0}=M_{1}=15$, and α=150, for example, $f_{0}=f_{1}=f$ = 6. When $M_{0}=M_{1}=15$, and α=200, $f_{0}=f_{1}=f$ = 2.

### 5.4. Post Optimization

So far, we have assumed that the backward reduction phase is carried out for each fragment semijoin in the optimal sequence. As described in Section 4.1, the values for which false positives are confirmed need to be sent back again and their corresponding tuples need to be inserted back to the reduced relation. In the backward reduction, if the matched joining attribute values are sent back with their counts, re-insertion would not be necessary at all. In doing so, the concern is the overhead of sending the count for each individual matched value. An efficient way of handling this problem is to delay the backward reduction of each fragment semijoin until the execution of all the fragment semijoins in the optimal sequence is completed, and to conduct the backward reductions all at once. For this, $R_{i}$ sends $R_{1-i}$ a message of the form: $\;n_{j}$, $n_{j+1}$, …, $n_{M_{i}}$, $V_{j}$, $V_{j+1}$, …, $V_{M_{i}}$ where $j=\lceil \text{α}/M_{1-i}\rceil$, $V_{j}$ is the set of matched joining attribute values (possibly including those from false positives) whose count is equal to *j*, and $n_{k}=\left|V_{k}\right|\;(\;k=j,\;j+1,\;\cdots$, $M_{i}$).

### 5.5. Query Optimization and Processing

**Figure 10:**
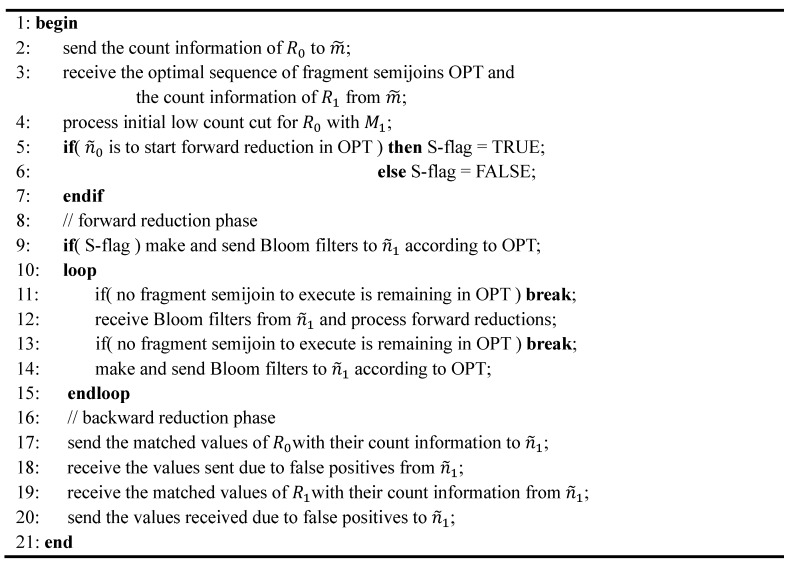
Protocol Executed by ${\tilde{n}}_{0}$ for Our Scheme.

In-network optimization of an iceberg join query *Q* is carried out in the sensor node $\tilde{m}$ located at the midpoint between ${\tilde{n}}_{0}$ and ${\tilde{n}}_{1}$. Thus, it is required for ${\tilde{n}}_{0}$ and ${\tilde{n}}_{1}$ to send the information necessary for the optimization to $\tilde{m}$. First, ${\tilde{n}}_{i}$ sends the count information of $R_{i}$ (*i.e*., $\left|R_{i}^{1}\right|$, ⋯, $\left|R_{i}^{M_{i}}\right|$) to $\tilde{m}$. ||A|| and $\left|D_{A}\right|$ are assumed to have already been sent to $\tilde{m}$ when *Q* was initially forwarded to ${\tilde{n}}_{0}$, ${\tilde{n}}_{1}$, and $\tilde{m}$. All the equations inSection 5 and Section 3.2 can be evaluated if the aforementioned information is available. The node $\tilde{m}$ generates the optimal sequence of fragment semijoins and sends it to ${\tilde{n}}_{i}$ with the count information of $R_{1-i}$ (i.e., $\left|R_{1-i}^{1}\right|$, ⋯, $\left|R_{1-i}^{M_{1-i}}\right|$). The count information of $R_{i}$ as well as the optimal sequence as described in Section 5.1 are represented as a short sequence of integers. Thus, the communication overhead for optimization could be very small. Now ${\tilde{n}}_{0}$ and ${\tilde{n}}_{1}$ are ready to execute the optimal sequence to fully reduce $R_{0}$ and $R_{1}$.The skeleton of the protocol executed by ${\tilde{n}}_{0}$ for our scheme is described in Figure 10. The one for ${\tilde{n}}_{1}$ is symmetrical.

## 6. Performance Evaluation of Our Scheme and SRJA

In this section, we compare the performance of our scheme and that of SRJA. We have implemented both of our scheme and SRJA, measuring the total number of packets transmitted among the sensor nodes and the total number of transmissions among the sensor nodes while the two schemes are executed. These two performance metrics are employed to compare the energy-efficiency of the two schemes. The number of packets transmitted is measured assuming that the network is IEEE 802.15.4-compliant. We also measured the ratio of joinable values transmitted over all the values transmitted. This metric is to compare the effectiveness of data filtering as well as the energy-efficiency of the two schemes. Finally, we present an analytical comparison of the total number of transmissions in the two schemes. All the procedures for the experiments were implemented in C and the experiments were conducted in a system of Windows 7 with an AMD Phenom II X4 945 Processor (3.0 GHz) and 4 GB memory.

### 6.1. Parameters

The network and query parameters in the experiments are summarized inTable 2. We have considered WSNs where sensor nodes are uniformly deployed. Each of the two regions for an inter-region iceberg join where a join operand relation resides is assumed to be a square consisting of *n* × *n* nodes. The distance between the coordinator nodes ${\tilde{n}}_{0}$ and ${\tilde{n}}_{1}$ of the two regions is set to 30 hops.

**Table 2:** Parameter Settings in the Experiments.

Parameter	Value
Size of a region (*n* × *n* nodes)	*n* = 5, 10, 15
The distance between ${\tilde{n}}_{0}$ and ${\tilde{n}}_{1}$	30 hops
The range of joining attribute values	1..10,000
The range of count	1..15
Epoch (*i.e.*, sampling interval)	10, 20, 30, 40, 50, 60 s(default: 30 s)
Query window size	3 h
Iceberg threshold (α)	50, 100, 150, 200

The default epoch (*i.e.*, sampling interval) of every sensor node is set to 30 s, and the size of the sliding window of the join query is set to 3 h as in the experiments with SRJA in [5]. After setting the join selectivity between $R_{0}$ and $R_{1}$, the values of the joining attribute in the iceberg join operand relations are randomly generated as an integer in the range [1,10,000] with a random distribution of their counts under the constraint that the highest count for a value could be 15. The iceberg thresholds considered are 50, 100, 150 and 200.

### 6.2. Experimental Results

**Figure 11:**
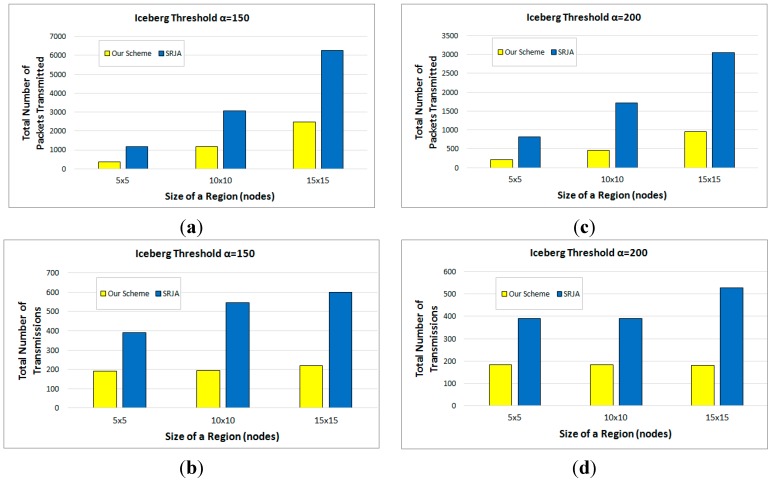
Performance Comparison with respect to Varying Sizes of a Region. (**a**) Total number of packets transmitted (α=150); (**b**) Total number of transmissions (α=150); (**c**) Total number of packets transmitted (α=200); (**d**) Total number of transmissions (α=200).

Each of the reported measurements in this subsection is the average out of 50 runs against different data values. The experimental results reveal that our scheme considerably outperforms SRJA. Figure 11a,b respectively compare the total number of packets transmitted and the total number of transmissions of the two schemes as the size of each region varies from 5 × 5 nodes to 15 × 15 nodes while α is set to 150. Figure 11c,d compare the same while α is set to 200. As the size of each region gets bigger, more data is collected at the sensor nodes and more tuples need to be processed in both schemes. Thus, more packets are transmitted. As for the number of transmissions, it also increases in SRJA. In our scheme, it is not so sensitive to the data volume because for a given α, the optimal sequence of fragment semijoins generated could include the similar number of semijoins. For the total number of packets transmitted, the average performance improvement with our scheme over SRJA is 63.58% (α= 150) and 71.97% (α= 200). For the total number of transmissions, the average performance improvement with our scheme over SRJA is 59.66% (α= 150) and 57.27% (α= 200).

**Figure 12:**
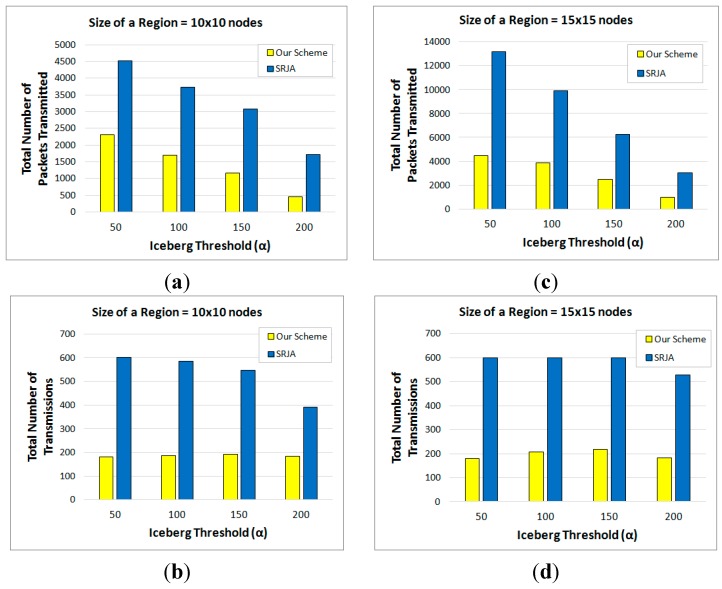
Performance Comparison with respect to Varying Iceberg Thresholds. (**a**) Total number of packets transmitted (10 × 10 nodes); (**b**) Total number of transmissions (10 × 10 nodes); (**c**) Total number of packets transmitted (15 × 15 nodes); (**d**) Total number of transmissions (15 × 15 nodes).

Figure 12a,b respectively compare the total number of packets transmitted and the total number of transmissions of the two schemes as α varies from 50 to 200 while the size of each region is set to 10 × 10 nodes. Figure 12c,d compare the same while the size of each region is set to 15 × 15 nodes. As α increases, the iceberg join query gets more selective. Thus, in both schemes, more tuples are filtered and the number of packets transmitted decreases. The number of transmissions turns out not so sensitive to the increase of α except for the case of α=200. In SRJA, the effect of subrange pruning results in the decrease of transmissions for larger α’s. In our scheme, on the other hand, the number of transmissions slightly increases from α=50 to 150, then decreases for α=200. These changes depend on the optimal sequence of fragment semijoins generated. The more semijoins are to be executed in the optimal sequence, the more transmissions would occur. For the total number of packets transmitted, the average performance improvement with our scheme over SRJA is 59.71% (regions of 10 × 10 nodes) and 64.07% (regions of 15 × 15 nodes). For the total number of transmissions, the average performance improvement with our scheme over SRJA is 63.98% (regions of 10 × 10 nodes) and 66.13% (regions of 15 × 15 nodes).

**Figure 13:**
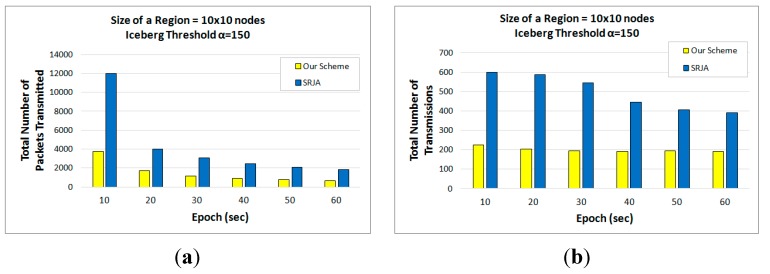
Performance Comparison with respect to Varying Epochs. (10 × 10 nodes, α=150). (**a**) Total number of packets transmitted; (**b**) Total number of transmissions.

Figure 13a,b respectively compare the total number of packets transmitted and the total number of transmissions of the two schemes as the epoch (*i.e.*, sampling interval) at each node varies from 10 s to 60 s while the size of each region is set to 10 × 10 nodes and α is set to 150. As the sampling rate gets higher, more data is collected at the sensor nodes and both schemes are supposed to transmit more packets. The number of transmissions also increases. However, SRJA turns out to suffer much more with higher sampling rates. For the total number of packets transmitted, the average performance improvement with our scheme over SRJA is 62.97%. For the total number of transmissions, the average performance improvement with our scheme over SRJA is 58.66%.

**Figure 14:**
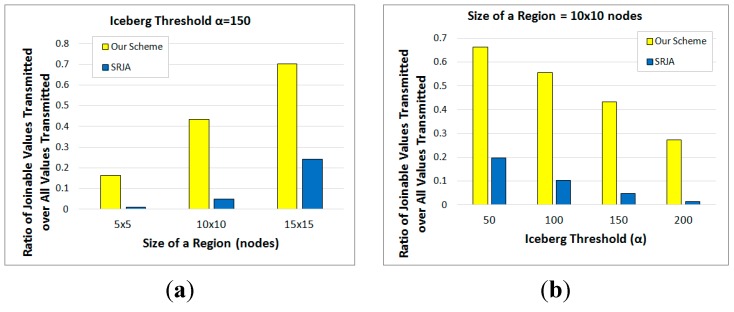
Comparison of the Ratio of Joinable Values Transmitted with respect to (**a**) varying sizes of a region (α=150); (**b**) varying iceberg thresholds (10 × 10 nodes).

Figure 14a compares the ratio of joinable values transmitted in the two schemes as the size of each region varies from 5 × 5 nodes to 15 × 15 nodes while α is set to 150. This ratio is defined as:

(12) $$\frac{\text{Total number of joinable attribute values }}{\text{Total number of join attribute values transmitted}}$$

Figure 14b compares the same as α varies from 50 to 200 while the size of each region is set to 10 × 10 nodes. This ratio gets lower as the query gets more selective or as more non-joinable values are transmitted. As the size of each region gets bigger, the number of tuples collected at the sensor nodes increases and the size of the iceberg join result also increases with a given α. Thus, this ratio increases in both schemes (Figure 14a). As α increases, on the other hand, this ratio decreases in both schemes because the iceberg join query gets more selective (Figure 14b). In Figure 14, this ratio in our scheme turns out to be significantly higher than that in SRJA. This means that the effectiveness of filtering out non-joinable values and the energy-efficiency in our scheme is much higher than that in SRJA.

The major reasons for the improvements are two-fold:

- In SRJA, the histogram-based value ranges are sent as a synopsis of the joining attribute values. In our scheme, a Bloom filter constructed from a count-based fragment is sent as a synopsis of the joining attribute values. The Bloom filter is more compact than the value ranges. Besides, the false positives are efficiently handled in the backward reduction phase of the 2-way fragment semijoins in our scheme.

SRJA is centered around checking the join predicate with the cardinality constraint as an additional condition. For each pair of matched ranges, if there exists at least one pair of tuples *t*_0_ $\in \;R_{0}$ and *t*_1_ $\in \;R_{1}\;$such that *t*_0_.count × *t*_1_.count ≥ α, the non-joinable tuples in either range cannot be filtered out, and recursive divisions of each of the two ranges into subranges are required. In contrast, our scheme is centered around the cardinality constraint with the join predicate as the secondary condition. Only the fragment semijoins between the fragments satisfying the cardinality constraint are carried out to filter non-joinable tuples. Besides, the reductions from the LCCs that result with the HCF strategy are effective.

### 6.3. Analytical Comparison of the Total Number of Transmissions

In this subsection, we present an analytical comparison of the total number of transmissions among the sensor nodes throughout the execution of SRJA and our scheme. In Figure 2 and Figure 10, the protocols executed by the coordinator node of a region for SRJA and our scheme are described. In each of the two schemes, let $X_{i,1-i}$ be the number of messages that ${\tilde{n}}_{i}$ sends to ${\tilde{n}}_{1-i}$ plus the number of messages that ${\tilde{n}}_{i}$ receives from ${\tilde{n}}_{1-i}$. Let $Y_{i}$ be the number of messages that ${\tilde{n}}_{i}$ sends to $\tilde{m}$ plus the number of messages that ${\tilde{n}}_{i}$ receives from $\tilde{m}$. Let $M_{x}=$ $X_{01}+X_{10}$ and $M_{y}=$ $Y_{0}+Y_{1}$. Then, the total number of transmissions in each scheme is $d\cdot M_{x}+\;\frac{1}{2}d\cdot M_{y}$ where *d* is the distance between ${\tilde{n}}_{0}$ and ${\tilde{n}}_{1}$ in hops.

#### 6.3.1. SRJA

Considering the protocol in Figure 2, we have:

(13) $$X_{i,1-i}=H_{i,1-i}+S_{i,1-i}+\sum_{k=1}^{N_{r}}\left(f_{k}^{J}\cdot J_{i,1-i}\;+\;f_{k}^{D}\cdot S_{i,1-i}\right)$$

$H_{i,1-i}$ denotes the number of messages ${\tilde{n}}_{i}$ sends to ${\tilde{n}}_{1-i}$ plus the number of messages ${\tilde{n}}_{i}$ receives from ${\tilde{n}}_{1-i}$ in processing the values with a high count (line 2 in Figure 2), $S_{i,1-i}$ denotes that in processing sparse subranges in the synopsis (line 4 or line 16), and $J_{i,1-i}$ denotes that in processing JOIN-tagged subranges (line 13). $f_{k}^{J}$ is 1 if the tagged synopsis received from $\tilde{m}$ includes at least one JOIN-tagged subrange. It is 0 otherwise. $f_{k}^{D}$ is 1 if the tagged synopsis includes at least one DIVIDE-tagged subrange. It is 0 otherwise. The sparse subranges could be generated after the synopsis is initialized (line 3) or after the DIVIDE-tagged subranges are divided (line 15). Finally, $N_{r}$ denotes the number of rounds needed until SRJA is terminated. It means how many times the loop (line 5 through line 18) is repeated either fully or partially (up to line 9 for breaking the loop). Thus, $N_{r}\;\geq 1$. Now $M_{x}$ is:

(14) $$\left(H_{01}+H_{10}\right)+(S_{01}+S_{10})+\sum_{k=1}^{N_{r}}\left(f_{k}^{J}\cdot (J_{01}+J_{10})\;+\;f_{k}^{D}\cdot (S_{01}+S_{10})\right)$$

Normally, $H_{01}=\;2$ because a 2-way semijoin is executed. ${\tilde{n}}_{0}$ first sends the values with a high count with their count information to ${\tilde{n}}_{1}$, and then receives from ${\tilde{n}}_{1}$ the list of joinable values as a result (line 2). ${\tilde{n}}_{1}$ does the same symmetrically. If these two symmetrical processes are conducted as an asymmetrical one, one message can be saved, and thus, the number of transmissions could be significantly reduced when the distance between ${\tilde{n}}_{0}$ and ${\tilde{n}}_{1}$ is long. We can let ${\tilde{n}}_{0}$ start the process by sending its data to ${\tilde{n}}_{1}$. When ${\tilde{n}}_{1}$ returns the result, it can send its data as well. Then, ${\tilde{n}}_{0}$ finally returns the result. In this way, $H_{01}+H_{10}=3$. Similarly, $S_{01}+S_{10}=3$. Meanwhile, $J_{01}+J_{10}=\;2$ because one 2-way semijoin in either direction but not the symmetrical two is enough in processing the JOIN-tagged subranges. Thus:

(15) $$M_{x}=6+\sum_{k=1}^{N_{r}}\left(2f_{k}^{J}\;+3f_{k}^{D}\right)$$

Meanwhile, $Y_{i}=\;2N_{r}$ because ${\tilde{n}}_{i}$ is to send the synopsis to $\tilde{m}$ (line 7) and receive the tagged synopsis from $\tilde{m}$ (line 8) in each round. Thus, $M_{y}=\;4N_{r}$. Let $T_{S}$ be the total number of transmissions in SRJA. Then:

(16) $$T_{S}=d\cdot (6+\sum_{k=1}^{N_{r}}\left(2f_{k}^{J}\;+3f_{k}^{D}\right)+2N_{r})$$

#### 6.3.2. Our Scheme

Considering the protocol in Figure 10, we have $X_{i,1-i}=\;$ $F_{i,1-i}+\;B_{i,1-i}$, where $F_{i,1-i}$ denotes the number of messages ${\tilde{n}}_{i}$ sends to ${\tilde{n}}_{1-i}\;$in the forward reduction phase (line 9 through line 15 in Figure 10), while $B_{i,1-i}$ denotes the number of messages ${\tilde{n}}_{i}$ sends to ${\tilde{n}}_{1-i}\;$ plus the number of messages ${\tilde{n}}_{i}$ receives from ${\tilde{n}}_{1-i}\;$ in the backward reduction phase (line 17 through line 20). Now $M_{x}$ is $\left(F_{01}+F_{10}\right)+(B_{01}+B_{10})$.

In executing the optimal sequence of fragment semijoins, $R_{0}$ and $R_{1}$ are supposed to take turns to play the role of reducer relation. Thus, the number of messages including the Bloom filters for a subsequence of fragment semijoins in the forward reduction phase is equal to the number of this turn overs denoted as $N_{t}$. For example, in the sequence of fragment semijoins in Figure 4, there are 5 turn overs with $R_{1}$ being the reducer relation at first in Figure 4b. Thus, $F_{01}+F_{10}=\;N_{t}$. With the same argument for why $H_{01}+H_{10}=3\;$in SRJA, $B_{01}+B_{10}=3$. Thus, $M_{x}=\;$ $N_{t}+3$.

Meanwhile, $Y_{i}=\;$ 2 because ${\tilde{n}}_{i}$ sends the count information of $R_{i}$ to $\tilde{m}$ once (line 2) and receive from $\tilde{m}$ the optimal sequence and the count information of $R_{1-i}$ once (line 3). Let $T_{O}$ be the total number of transmissions in our scheme. Then:

(17) $$T_{O\;}\;=d\cdot \left(N_{t}+5\right)$$

From (Equations (16) and (17)), $T_{O}<$ $T_{S}$ if $N_{t}<$ $\overset{N_{r}}{\underset{k=1}{\sum }}\left(2f_{k}^{J}\;+3f_{k}^{D}\right)+2N_{r}+1$. For the comparison, let us not consider the restricted case of $N_{r}=1$. This case happens in SRJA only when at least one of $R_{0}$ and $R_{1}$ has very small number of tuples and their join attribute values are sparsely distributed. Such a case is not of much interest. Now since $N_{r}\;\geq$ 2, we have $\overset{N_{r}}{\underset{k=1}{\sum }}\left(2f_{k}^{J}\;+3f_{k}^{D}\right)≅5(N_{r}-1)$. The reason for this is as follows: in Figure 10, we note that $f_{N_{r}}^{J}=f_{N_{r}}^{D}=0$ for the final round. In each of the interim rounds, we note that $f_{k}^{D}=1$ because without any DIVIDE-tagged subrange the next round except the final one would not be necessary. In each of the interim rounds, it is not necessarily that $f_{k}^{J}=1$. Assuming that normally it holds, $T_{O}<$ $T_{S}$ iff $N_{t}<$ 7$N_{r}-4$. The values of $N_{t}$ and $N_{r}$ depend on several parameters such as the number of tuples, distribution of joining attribute values, their count distribution, iceberg threshold, effectiveness of non-joinable value filtering, and so on. In the experiments in the previous subsection, it turns out that $N_{t}<$ 3 while $N_{r}\;\geq$ 2 on average. For such values, $T_{O}<$ $T_{S}$ as shown by the measurements in the experiments.

## 7. Related Work

In-network processing of joins in WSNs has received much attention [3]. The state-of-the-art techniques deal with various types of join queries with different types of spatio-temporal characteristics. Spatially, the type of join that has received so far the most attention is the inter-region join where each of the join operand relation is stored at a region of WSNs [2,6,19,20,21,22,23,24]. The pair-wise joins where pairing of sensor nodes for join could be determined by predicates are investigated in [25,26]. Temporally, one-shot join against a static join operand relations are dealt with in [2,23,27], while the join submitted as a continuous query against the streaming join operand relations are dealt with in [19,20,21,24,25,26,28,29]. As for the type of join predicates, equijoin queries are considered in [2,19,20], while theta-joins are considered in [20,21,22,23,24,25,26,27,28,29,30].

These techniques mostly adapt the conventional join implementations to WSNs (nested-loop join [20,21,22,23], hash join [2,19,21], and the sort-merge join [19]). The semijoins and 2-way semijoins are also employed and adapted to WSNs for filtering of non-joinable tuples [2,23,24,27,29]. In [28,30], on the other hand, a filtering approach is proposed rather than adapting the conventional join algorithms to WSNs. The cost-based optimizations are investigated in [21,22,23,24,25,26,28].

In [21], a technique called *Distribute-Broadcast Join* adapted from the nested-loop join is proposed. The main contribution of this work is the cost-based selection of optimal join region in WSNs. In [22,23], *Mediated Join* also adapted from the nested-loop join is proposed, where cost-based selection of inner and outer relation for nested-loop join is investigated. In [20], distributed algorithms for indexed nested-loop join and hash join are proposed. In the former, a technique of dynamically creating and using a distributed B+ tree in WSNs is developed. In the latter, a technique of partitioning and joining tuples with geographic hashing is investigated. In [25,26], a *pair-wise join* between any two sensor nodes is investigated. Multiple routing trees are employed and cost-based join initiation for long-running join query is proposed. Also, the issue of adaptive and cost-based re-optimization against the changes of sampled data is dealt with. In [2], *Synopsis Join* adapted from hash join as well as semijoin is proposed. It employs geographic hashing for partitioning and filtering of the joining attribute values and optimally determines the nodes of the final joins for each matched value. In [29], *Two-Phase Self Join* where one join operand relation is fully reduced with a semijoin is proposed. To process a join query, it employs a query decomposition technique assuming that the selection predicate on one relation is highly selective. In [27], *SENS-Join* based on a 2-way semijoin is proposed. It handles a general type of join predicates on multiple attributes, using a quad-tree as a multi-dimensional join filter based on Z-ordering. In [19], *PEJA* adapted from hash join as well as sort-merge join is proposed. In WSNs, physical sorting of the tuples distributed over the sensor nodes is infeasible. Thus, it conducts logical sort of tuples through division of joining attribute value range, partitioning and filtering tuples with geographic hashing. In [28,30], algorithms using the join filters are proposed for continuous queries on multiple attributes. At each sensor node involved in the join query processing, a join filter is installed for each joining attribute and only those tuples whose join attribute values pass the relevant filters are sent to the base station.

Other issues addressed by the state-of-the-art includes routing protocols, query dissemination, join initiation, involvement of the base station in in-network processing, collection of metadata for continuous joins [3].

In [18,24,31], in-network join processing where the Bloom filter is employed in WSNs are addressed. In [18], for a join between an external relation and the virtual relation in WSNs, a technique where a Bloom filter constructed with the joining attribute values of the external relation is injected into the network is investigated. In [24], a technique where the Bloom filter is transmitted instead of the joining attribute values in in-network computation of the two semijoins $R_{0}$ $⋉R_{1}$ and $R_{0}$ ⋊ $R_{1}$ to answer a join $R_{0}$ ⋈ $R_{1}$, is proposed. In this technique, when the Bloom filter constructed for $R_{0}$ is disseminated through the routing tree of $R_{1}$, it is selectively forwarded to the subtrees of the routing tree such that the cost of sending it is always compensated in reduction. The optimal solution in such a selective forwarding is proposed. In [31], an extension of the Bloom filter called Window Bloom filter (WBF) is devised to support a general join query with a time window. The proposed technique represents join attribute values in a compact way with the WBF, and saves the energy consumption by not sending the redundant join attribute values.

As described in Section 3.3, in SRJA [6], the value range instead of the joining attribute values is sent as a histogram-based synopsis. Transmitting the value ranges instead of the joining attribute values is considered for in-network join processing [18]. In [2], a technique using a histogram-based synopsis of the join operand relations coupled with geographic hashing is proposed. However, the considered join in these techniques is not an iceberg join.

The iceberg query was first introduced in [32]. It is defined as a query that retrieves aggregate values above some specified threshold in the applications such as data warehousing, data mining, information retrieval, and so on. Iceberg query processing over distributed data was also investigated [33,34,35]. However, the join query was not dealt with in these work. In [7], an iceberg distant join in spatial databases was investigated. In this work, the type of the join query is different from the one we consider in this paper in that the cardinality constraint is only on one join operand relation. That is, when the iceberg threshold is α, the tuple of one relation that is joined with more than α tuples of the other relation is qualified for the query.

## 8. Conclusions

In this paper, we investigated an alternative approach to processing an iceberg join in WSNs, and described an optimized scheme. In the previous approach, the join predicate is checked first, and the cardinality constraint is checked next. In our approach, the order is reversed. Our scheme refers to the aggregate count of each of the joining attribute values, logically fragmenting the relations on the counts. Based on the fragmentation, it generates the optimal sequence of 2-way fragment semijoins using a Bloom filter as a synopsis of joining attribute values in filtering non-joinable tuples. A detailed set of experiments showed that our approach is substantially superior to the previous one.

As a future work, we plan to extend our scheme for more complicated cases. First, the two regions producing the virtual relations to be joined could partially or fully overlap with each other. Secondly, more than two regions are involved in multiple joins. Thirdly, the correlations among the sensor readings along the neighboring *n* regions are to be monitored with an *n*-way join.
